# Graph drawing using tabu search coupled with path relinking

**DOI:** 10.1371/journal.pone.0197103

**Published:** 2018-05-10

**Authors:** Fadi K. Dib, Peter Rodgers

**Affiliations:** 1 School of Computing, University of Kent, Canterbury, Kent, United Kingdom; 2 Computer Science Department, Gulf University for Science and Technology, Hawally, Kuwait; Universita degli Studi di Catania, ITALY

## Abstract

Graph drawing, or the automatic layout of graphs, is a challenging problem. There are several search based methods for graph drawing which are based on optimizing an objective function which is formed from a weighted sum of multiple criteria. In this paper, we propose a new neighbourhood search method which uses a tabu search coupled with path relinking to optimize such objective functions for general graph layouts with undirected straight lines. To our knowledge, before our work, neither of these methods have been previously used in general multi-criteria graph drawing. Tabu search uses a memory list to speed up searching by avoiding previously tested solutions, while the path relinking method generates new solutions by exploring paths that connect high quality solutions. We use path relinking periodically within the tabu search procedure to speed up the identification of good solutions. We have evaluated our new method against the commonly used neighbourhood search optimization techniques: hill climbing and simulated annealing. Our evaluation examines the quality of the graph layout (objective function’s value) and the speed of layout in terms of the number of evaluated solutions required to draw a graph. We also examine the relative scalability of each method. Our experimental results were applied to both random graphs and a real-world dataset. We show that our method outperforms both hill climbing and simulated annealing by producing a better layout in a lower number of evaluated solutions. In addition, we demonstrate that our method has greater scalability as it can layout larger graphs than the state-of-the-art neighbourhood search methods. Finally, we show that similar results can be produced in a real world setting by testing our method against a standard public graph dataset.

## 1. Introduction

Graph drawing is the process of transforming a graph into a visual representation that is called a graph layout [1]. The graph layout depends on different aesthetic measures that could give a better understanding of graphs. Such measures include minimizing edges crossings, uniform edge length, maximizing node-to-node and node-to-edge occlusions, maximizing graph symmetry, maximizing angular resolution, and others [1, 2, 3, 4]. These measures can be combined to form a multi-criteria weighted sum objective function that measures the quality of a graph and then optimized by search based methods (optimization methods).

Search based methods can be placed into two categories according to the number of solutions examined at the same time: neighbourhood search methods and population based methods. While neighbourhood search methods work on a single solution at a time, population based methods evolve a set of points in the search space [5]. These methods can produce good solutions, but they have great potential for improvement. For example, in neighbourhood search methods, simulated annealing adds an element of non-determinism in order to escape from local optima in the search space. This slows down the performance of the algorithm since this stochastic behaviour means that a large number of iterations can be required to reach a good solution [2]. Hill climbing is generally faster in reaching a final layout, but the final result is not always the best as it is more likely to get trapped in a local optima [6]. Population based methods such as genetic algorithms typically have an even slower rate of convergence compared to simulated annealing and hill climbing as they make a wider search of the problem space. In addition they often require large memory to maintain the population and can require additional algorithms to spread the solutions [7].

Many graph layout algorithms in the literature use neighbourhood search based methods for drawing multi-criteria graph layouts with Simulated Annealing (SA) [2, 8, 9, 10] and Hill Climbing (HC) [3, 6, 11], and single-criterion graph layouts with Tabu search (TS) [12] and Path Relinking (PR) [13]. On the other hand, population based methods have also been used in drawing multi-criteria graph layouts with genetic algorithms [14, 15, 16, 17].

Neighbourhood search based layout is frequently used in graph drawing. It is used to optimize different quality measures that are combined as a single weighted sum objective function. However, such methods work slowly as the process of recalculating the metrics is repeated a large number of times during the search process. Here we propose a novel neighbourhood search based graph drawing algorithm and compare it with other such approaches. We show that our approach improves on the current state-of-the-art in neighbourhood search for graph drawing.

Another popular type of layout is the class of Force-directed approaches. These differ considerably from search based methods. Here, interactions between nodes are applied such as the attraction of connected nodes and repulsion of disconnected nodes, where the method attempts to find an equilibrium layout [18, 19, 20, 21]. In addition, systems such as Pajek draw large networks using spring embedders and eigenvectors [22]. However aesthetics can only be indirectly coded in force directed approaches, whereas search based methods have the advantage of allowing tuneable combinations of metrics to meet user preferences.

The scope of the research described in this paper is to improve the efficiency and effectiveness of drawing general graph layouts with undirected straight lines based on a weighted sum multi-criteria optimization. The main goal of our work is concerned with developing a new graph drawing search method based on tabu search and path relinking. To our knowledge, these methods have not been used before to lay out general graphs with multi-criteria optimization.

Since our method belongs to the category of neighbourhood search methods, we compare it against hill climbing and simulated annealing as these are well-known neighbourhood search methods that have been frequently applied to graph drawing.

In this paper we show that tabu search alone outperforms hill climbing, but not simulated annealing, we then show that when tabu is combined with path relinking it outperforms simulated annealing. The tabu search algorithm outperforms hill climbing in minimizing the value of the objective function and the number of evaluated solutions used to draw a graph layout. The addition of applying path relinking within the tabu search procedure speeds up the identification of good solutions and outperforms simulated annealing by producing graph layouts with better values of objective function. We also demonstrate that when targeting a particular value of an objective function, the combination of tabu search and path relinking achieves the goal in a smaller number of evaluated solutions.

Path relinking integrates intensification and diversification strategies [23]. This approach generates new solutions by exploring paths that connect high quality solutions (elite solutions from the reference set) by starting from one of these solutions, called the initiating solution, and generating a path in the neighbourhood space that leads toward another solution, called the guiding solution. Note that the initiating and the guiding solutions represent the starting and the ending points of the path. This is accomplished by selecting moves that introduce attributes contained in the guiding solutions [13]. A crucial difference between evolutionary algorithms, such as genetic algorithms, and path relinking is that the former uses a factor of randomness to create offspring from parent solutions, whereas the latter uses systematic and deterministic rules to combine elite solutions. The main principle of its deterministic behaviour is the gradual introduction of attributes from the guiding solution to intermediate solutions. These attributes should have fewer characteristics from the initial solution and more characteristics from the guiding solution as the search moves along the path [24]. Path relinking has been considered to be particularly appropriate to tabu search as it allows for “tunnelling” through infeasible regions formed from the tabu list [25].

In a previous paper [26], we described an initial attempt to use a tabu search based approach for graph drawing and we compared it with hill climbing only. The method searches for the best positions for the nodes that minimize the value of the objective function, and draws a nice graph layout accordingly. Tabu search forbids moves that have been previously examined which may be considered poor potential solutions, making it a more effective layout method than hill climbing. In this paper, we widen the comparison to include simulated annealing. We also extend and improve our preliminary work [26] by coupling tabu with path relinking. This combined approach considerably improves the effectiveness of the search. We also conduct a more thorough comparison. Here we use four different neighbourhood search based methods: hill climbing, simulated annealing, tabu search and tabu search with path relinking. In addition, we change the criterion of comparison between the methods to the number of evaluated solutions calculated by the drawing algorithm (as this is a machine independent criterion) instead of using the algorithm’s execution time which was used in the preliminary work. We only present an execution time comparison when we test the scalability of the methods which was not tested in our previous work. In this case, we use execution time to give a realistic idea of run time for applying the methods. Statistical significance tests that confirm the results of our experiments are also included in this paper unlike our work in [26]. The code and data related to this research can be accessed at Dryad digital repository: doi:10.5061/dryad.k082rv8.

The rest of this paper is organized as follows: Section 2 describes some background in search based techniques; Section 3 describes our method that couples tabu search with path relinking; Section 4 describes parameters tuning process along with experimental results for applying hill climbing, simulated annealing, tabu search, and tabu search with path relinking on random graphs, and for testing the scalability of our method; Section 5 describes experimental results of applying the same approaches on real world public graph datasets; Section 6 discusses and analyses the results; finally, in Section 7 we give our conclusions in addition to directions for future work.

## 2. Background

Graph drawing is the process of placing nodes and edges in order to form clear and understandable layouts. However, this process is a challenge as it depends on what we consider as a nice graph (graph aesthetics) and the efficiency of its automated implementation. Graph drawing aesthetics are quality measures which determine the readability and usability of graphs. A good layout can deliver information clearly whereas a poor layout can mislead [27]. An objective function comprising metrics in a weighted sum can be used to quantify the quality of the graph layout and so be used within search based layout mechanisms. Typical criteria includes: minimizing edge crossings, minimizing edge bends, uniform edge length, maximizing graphs symmetry, maximizing node-to-node and node-to-edge occlusions and maximizing angular resolution of incident edges [2, 3, 4]. However, it is computationally expensive to find a minimum objective function’s value as the measurements can be time consuming to calculate, and the objective function is required to be determined for every layout examined. Since the overall objective function might include both continuous and discrete functions, some general search based approaches, such as neighbourhood search methods like simulated annealing and hill climbing, and population based methods such as genetic algorithms, have been used in order to find a good value of the objective function.

Simulated annealing was an early search based method to be applied to the graph layout problem [2]. It was used to draw undirected graphs with straight line edges. The original algorithm produces nice graph layouts for small sized graphs. However, it does not perform well for larger graphs. This approach models the physical process of heating a material and then slowly cooling the temperature to decrease defects, so minimizing the system energy. The method tries to escape from local minimum to global minimum by applying uphill moves (moves that worsen, rather than improve, the temporary solution) with decreasing probability as the search progresses. A variation of the approach uses gradient descent [8]. The gradient vector of the objective function represents the direction in which the node should move to increase the value of the objective function. However, this method is slow when being applied on large graphs and it has some challenges. For example, the objective function needs to be expressed explicitly in terms of coordinates as its derivative must be found. Some important criteria, such as minimizing edge crossings, are not continuous and so cannot be modelled with gradient decent.

Hill climbing is another search based approach that has been used in the field of graph drawing to minimize edge crossings [11]. It is a simpler and a faster search approach than simulated annealing as no uphill moves are made, but, as a result, tends to get trapped in poor local optima.

A genetic algorithm approach for drawing graphs under a number of visual constraints was proposed in [14, 15]. The proposed algorithm produces graph layouts with good quality. It can be easily adapted to take new layout aesthetics into account. However, the major problem is its slow rate of convergence.

Tabu search is a neighbourhood search based method proposed by Glover [28] for finding good solutions to combinatorial optimization problems. It is a neighbourhood search based procedure that uses a memory structure while it carefully explores the neighbourhood of each solution as the search progresses to avoid getting trapped in a local optima. It proceeds on the assumption that there is no value in choosing an inferior solution unless it is necessary, as in the case of escaping from a local optimum [29]. It improves the efficiency of the searching process by storing a tabu list of local solutions. This is used to restrict the search by forbidding moves to some poor neighbour solutions that have already been visited [30]. An additional feature of tabu search is applying intensification and diversification. It might be useful to intensify the exploration in some region because it may contain a high incidence of acceptable solutions. This can be obtained by introducing a new term in the objective function that assigns a high priority to solutions in the relevant region. Diversification is responsible for moving the exploration process over different regions of the search space. For example, the objective function can be adjusted so that it penalizes solutions which are close to the current one. Thus, tabu search can induce an interplay between intensification and diversification that is intended to form a dynamic and aggressive search strategy [12].

Tabu search has shown good results for generating approximate solutions to NP-hard problems in a reasonable amount of time [31, 32]. It has also produced comparably fast solutions in some graph theory applications such as graph partitioning [29, 33], as well as for special graph layout problems such as straight line crossing minimization [13, 34], and the bipartite graph drawing problem [12]. Tabu search has also outperformed many existing heuristics for solving the vehicle routing problem [35, 36, 37]. Tabu search was introduced for general graph drawing in our previous paper [26]. The experimental results on random graphs showed that our tabu search based approach was faster than hill climbing with good quality graph layouts. However, the performance comparison was not conclusive as it was based on the algorithm’s execution time, rather than the more accurate measure of number of evaluated solutions.

Path relinking is another neighbourhood search based method that was proposed as an approach to integrate intensification and diversification strategies [23, 38]. It is a relatively new approach that has been applied on several computational problems with a great success. The aim of path relinking is to introduce attributes of the guiding solution into solutions obtained by moving away from the initial solution in a systematic manner where similarities and differences in the structure of the initial and guiding solutions are properly identified.

Any path relinking implementation must include the following three components [24]:

- Building the reference set,
- Choosing the initial and the guiding solutions,
- Constructing a neighbourhood structure for moving along paths between initial and guiding solutions.

Using path relinking periodically in a search procedure is intended to speed up the identification of good solutions. Combining tabu search with path relinking is motivated by the desire to tunnel through blocked off areas created by the tabu solutions [25]. Solving a vehicle routing problem using tabu search with path relinking was able to generate better solutions compared to the traditional tabu search approach alone [24]. Tabu search and path relinking were also used to address the job shop scheduling problem and produced competitive results compared to alternative state of the art algorithms [39].

## 3. Our method

This section describes our graph drawing method based on tabu search coupled with path relinking. First we describe the tabu search procedure, then we detail how the path relinking is integrated into it.

**Fig 1:**
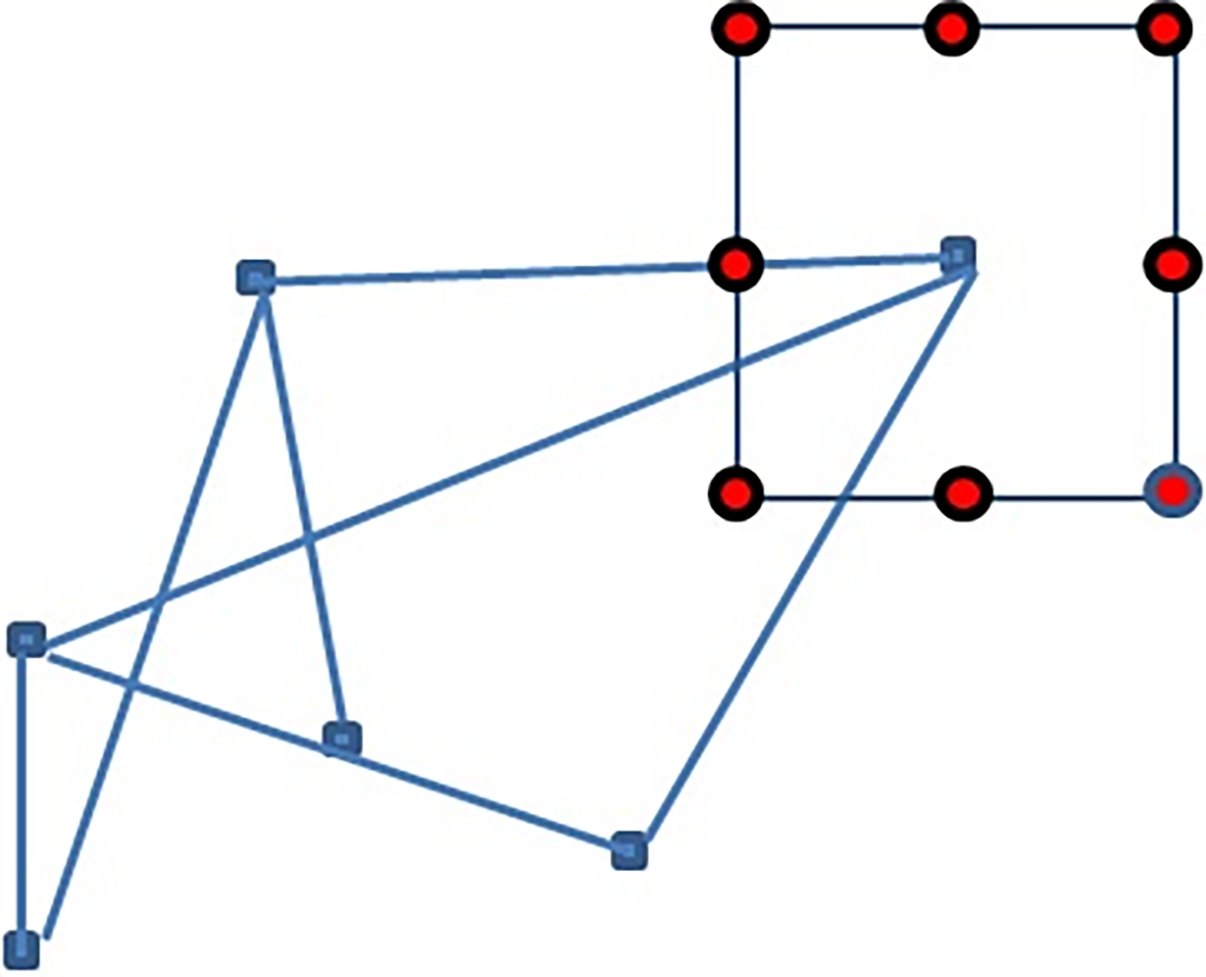
Points around the square where our method searches for candidate solutions.

In this work, we use a systematic exploration for local search space. For each node, we search the points (candidate solutions) around a square centred on the node at a given distance, as shown in Fig 1. Eight points around the square are checked (up, down, left, right, and the four corners). We compute the objective function’s value at each candidate solution, and we select the candidate solution that gives the lowest objective function’s value. In the case that there are multiple candidate solutions that share the lowest value, we select the first encountered candidate solution starting from the right point around the square and move along the points of the square in a clockwise direction. This is also how the objective function tie-breaks are resolved in the other methods.

Note that, using a geometric shape for defining a search space in the field of graph drawing was used earlier in [2, 3] where a circle and a rectangle had been respectively used. However, since evaluating the value of a multi-criteria objective function is a lengthy process, we restricted the movements to eight points only to avoid the long execution time for re-evaluating the value of the objective function with a large number of evaluated solutions. We used the same neighbourhood searching strategy with all the methods included in this research in order to make a fair comparison. This searching strategy can be easily adjusted with our implementation by increasing the number of repetitions from eight points to any larger number, but the execution time would be significantly longer.

In the tabu search procedure, we first compute the value of the objective function for the initial graph layout. Then, the following steps are performed for a set number of iterations (maxIterations): for each node, we search the points around a square as described above. The ratio of the objective function values for the candidate solution and the current solution is computed at each point around the square. Solutions with ratios above or equal to a predefined threshold value (tabuCutOff) are considered to be tabu moves and are stored in a tabu list. We then move the node to a neighbouring point which is not in the tabu list and its objective function value is minimal. Then the current location is added to the tabu list. Note that the new solution might not be better than the current solution. In case all eight candidate solutions surrounding the current solution are in tabu list, the intensification and the diversification processes are used. The search intensification process is as follows: after a chosen number of iterations (intensifyIterations), the square size centred on the node is reduced and the tabuCutOff value is decreased by a set value (intensifyCutOff) by calling function SmallerSquareSize() and function SmallerTabuCutOff() respectively as shown in Algorithm 1. The diversification process consists of updating the tabu list by removing old solutions from the list after a number of iterations (tabuDuration).

Our objective function follows a standard approach for search based graph drawing methods. We implemented four common metrics for measuring the quality of the graph similar to those used in [2, 3]. These represented the aesthetics of: spreading the nodes out evenly on the drawing space (node-node occlusion), making uniform edge lengths, minimizing edge crossings, and improving angular resolution (maximizing the distance between incident edges).

Spreading the nodes out evenly on the drawing space means that the distances between nodes should be maximized. This criterion was measured using the following formula:

$$\sum_{i\in V}\sum_{j\in V}\frac{1}{{d_{ij}}^{2}}$$ (1)

where *d*_*ij*_ represents the Euclidean distance between two nodes *i* and *j*, and *i* ≠ *j*.

Unifying the length of the edges was computed by defining a specific length (*len*), then all the edges would be adjusted to reach the required length using the following formula:

$$\sum_{e\in E}{\left(e-len\right)}^{2}$$ (2)

where *E* is the set of edges.

In order to minimize the number of crossing edges, we only needed to find the number of edge intersections and we tried to minimize that number in each iteration of the optimization process.

The last metric, the angular resolution, was computed by maximizing the distance between incident edges using the following formula:

$$\sum_{v\in V}\sum_{\{e_{1},e_{2}\}\in E}\left|\frac{2\pi }{\deg\left(v\right)}-\theta (e1,e2)\right|$$ (3)

where *deg*(*v*) denotes the degree of a node *v*, and θ(*e1*,*e2*) is the angle in radians between two adjacent edges *e1* and *e2* incident to node *v*.

All these metrics contribute in the graph quality objective function which is computed as follows:

objectiveFunction=w1*m1+w2*m2+w3*m3+w4*m4 (4)

where *w*_*i*_ and *m*_*i*_ are the weight and the measure for the criterion *i* respectively.

Similar multi-criteria objective functions have been previously used in [2, 3]. The metrics used are well-known and have been shown to represent a quality of a graph layout [27]. The problem in a multiple objective optimization function is that the value of a specific measure may dominate the others. Therefore, we applied a normalization process to ensure that the value of each measure is between 0 and 1.

We cannot determine unified weights that work well for all graphs with any size, and indeed weights can vary according to application area and user preference. The goal of our research is to develop an improved optimizer for general undirected graphs, rather than concentrate on generating the best possible layout. Hence, we take the approach of assuming all criteria are equally important and assign the value 1 to all weights.

Re-computing the objective function for each node move is a time consuming process. Therefore, we implemented a system that caches the results for each node and edge. When calculating objective function, we only compute the values that might change when a node is moved.

We couple our tabu search procedure with path relinking to intensify the search within a specific space of elite solutions as described in Algorithm 1. The path relinking procedure is called within the tabu search procedure every fixed number of iterations (intensifyIterations). Building a reference set of elite solutions is the first step in path relinking. This has a maximum size (refSize) and contains no redundant solutions and is recommended to be relatively small [24]. Initially, the solutions which are produced by the tabu search procedure are added to the reference set. A solution is directly added to the reference set as long as the set is not full. However, once the reference set becomes full, a solution will replace the worst solution in the set when any of the following criteria is satisfied:

(a) Quality: the objective function’s value of the added solution is better (smaller) than the objective function’s value of the best solution in the reference set. This is performed by the Quality() function in Algorithm 1.
(b) Diversity: the objective function’s value of the added solution is better (smaller) than the objective function’s value of the worst solution in the set, and it is dissimilar to the solutions in the set. The dissimilarity measure is computed as follows: we define $D_{s}^{b}$, the level of dissimilarity between solution *s* and the best solution *b*, as the sum of distances between the corresponding nodes in the two graph layouts. This is performed by the Diversity() function in Algorithm 1.

We also define the median position of all solutions *x* ∈ *refSet* relatively to the best solution *b* as:

$$median\;position=\frac{\sum_{x\in refSet}^{x\neq b}D_{x}^{b}}{|refSet|-1}$$ (5)

where |*refSet*| denotes the number of solutions in the reference set. A solution *s* is included in *refSet* if its objective function’s value is better than the objective function’s value of the worst solution in *refSet* and its level of dissimilarity exceeds the median.

**Fig 2:**
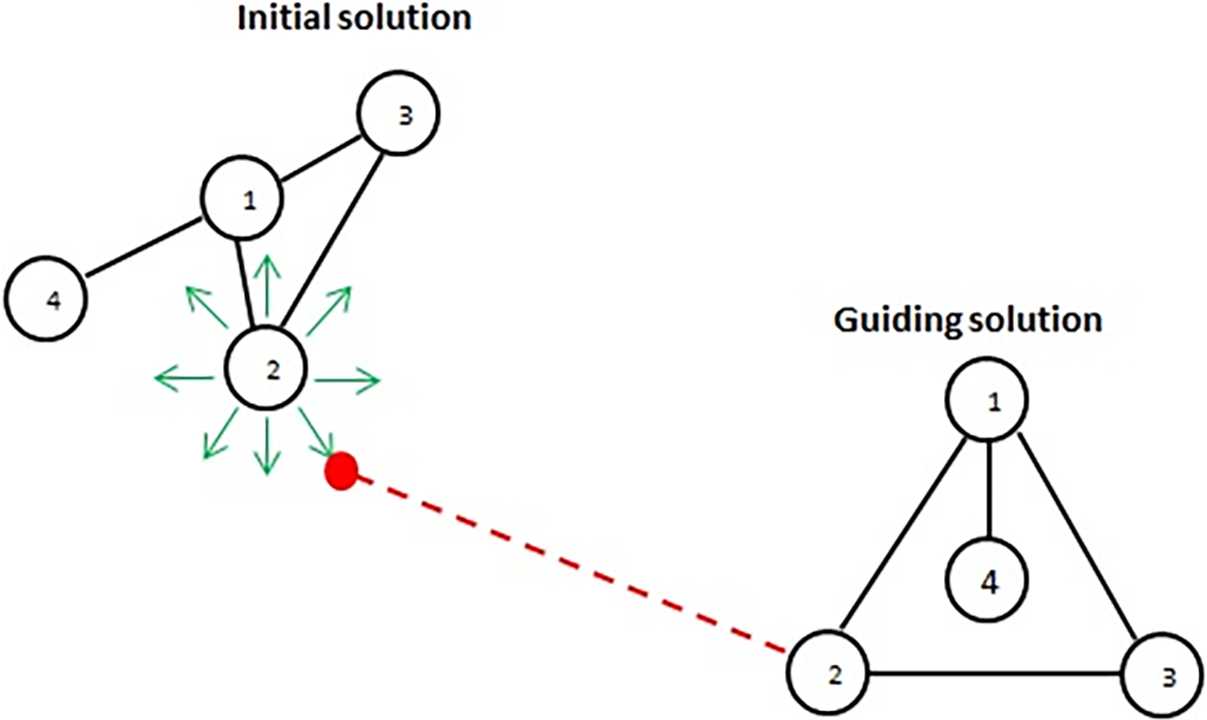
Our path relinking strategy in moving from initial solution to guiding solution.

When the path relinking procedure is called, the following steps are performed for a set number of iterations (PRmaxIterations) as long as the reference set has more than one solution (see Algorithm 2): firstly, we select two solutions from the reference set (initial and guiding solutions). There are different ways for selecting these two solutions [24, 39, 40] as we show later in this section. In this paper, the guiding solution is always of a better (smaller) objective function’s value than the objective function’s value of the initial solution. Secondly, we remove the initial solution from the reference set as its path to the guiding solution will be explored. Thirdly, we call function MoveAlongPath(), which moves on a path from the initial solution toward the guiding solution and vice versa to generate intermediate solutions (as this has produced better results in other applications [24] compared to moving in one direction only). These intermediate solutions should become closer to the guiding solution (i.e. contain more attributes from the guiding solution and less attributes from the initial solution). In our algorithm, for each node in the initial solution, we visit the 8 positions around a square (same positions described previously) of a predefined size (pathSqrSize) and compute the Euclidean distance from each position to its corresponding node in the guiding solution as shown in Fig 2. We select the position with the shortest Euclidean distance. Its objective function’s value is computed along with its dissimilarity level, and we update the reference set, by calling function UpdateReferenceSet(), if the new solution satisfies quality and dissimilarity measures. The movement along the path stops when an intermediate solution reaches the guiding solution or when the length of the path reaches a predefined value of a maximum length (pathLength). Note that, as we generate intermediate solutions, we use the tabu search memory-based list to avoid previously visited solutions.

**Algorithm 1.** Tabu Search/Path Relinking Algorithm

  **Given**:

    Connected Graph G(V,E): V is a set of nodes and E = (VxV) is a set of edges.

      initialSquareSize: predefined square size where tabu search candidate solutions are located on its border.

      squareReduction: predefined value which represents the rate of reduction for the size of the square.

    maxIterations: predefined maximum number of iterations of the tabu search drawing algorithm.

    initialCutOff: predefined minimum value that determines whether a move is tabu or not.

      intensifyCutOff: predefined value which represents the rate of reduction for current cutOff value.

    intensifyIterations: predefined number of iterations in which the tabu search searching process starts to intensify.

    duration: predefined number of iterations in which a move should remain in the tabu list.

      refSize: predefined size for the maximum number of solutions that can be added to the reference set of path relinking.

1: initialize tabuSet and refSet to {}

2: initialize cutOff to initialCutOff

3: for each i in [1, maxIterations] do:

4:     for each node v ∊ V do:

5:                 initialize candidates to {}

6:                 compute current objective function at node v (currentSolution)

7:             move v to the 8 positions around the square

8:             compute the objective function at each position (candidateSolution)

9:             if candidateSolution ∉ tabuSet and ratio of candidateSolution to currentSolution ≤ cutOff then:

10:                     add candidateSolution to candidates

11:             else

12:                     add candidateSolution to tabuSet

13:     choose a solution from candidates with the minimal objective function’s value (chosenSolution)

14:     if number of solutions in refSet < refSize or Quality (chosenSolution) or Diversity (chosenSolution) then:

15:             add chosenSolution to refSet

16:     after each intensifyIterations do:

17:             apply path relinking procedure

18:             reduce square size: SmallerSquareSize(squareSize, squareReduction)

19:             reduce cutoff value: SmallerTabuCutOff(intensifyCutOff)

20:             remove tabu solutions from tabuSet which spent a period of time (duration)

**Algorithm 2.** Path Relinking Procedure

  **Given**:

    PRmaxIterations: predefined value of the number of iterations to repeat the path relinking procedure

    pathSqrSize: predefined square size where path relinking candidate solutions are located on its border.

    pathLength: predefined value representing the maximum length of the path.

    accelerationPeriod: predefined number of iterations required for updating the searching step-size.

    accelerationRate: predefined value representing the rate of decreasing the searching step-size.

1: i = 0

2: while i < PRmaxIterations and Size(refSet) > 1 do:

3:         select source and destination solutions from refSet

4:         candidateLayout1 = MoveAlongPath(source, destination, pathLength, pathSqrSize, accelerationRate, accelerationPeriod) /* forward path */

5:         candidateLayout2 = MoveAlongPath(destination, source, pathLength, pathSqrSize, accelerationRate, accelerationPeriod) /* backward path */

6:         UpdateReferenceSet(Min(candidateLayout1, candidateLayout2))

7:         i = i + 1

Different selections for the initial (source) and guiding (destination) solutions affect the quality of graph layouts drawn by the path relinking procedure. There are five different variations for the selection mechanism of the source solution and the destination solution from the reference set [24]:

(a) The worst and the best elite solutions.
(b) The best and the second best elite solutions.
(c) Random selection of elite solutions.
(d) The best elite solution and the most distant elite solution to the best. In our application, the distance between two layouts can be computed as the summation of Euclidean distances between the corresponding nodes in the two layouts as described in Diversity() function used in Algorithm 1. The most distant solution is the one with the maximum summation of distances to the best elite solution (i.e. the most distant solution = *s* such that *s* ∈ *refSet* and satisfies the formula:
  $$max\;\sum_{s\in refSet}^{s\neq b}D_{s}^{b}$$ (6)
  where *b* is the best solution in *refSet* and $D_{s}^{b}$ is the level of dissimilarity between solutions *s* and *b*).
(e) The two most distant elite solutions.

We tested these five different variations on random connected graph datasets. The results showed that, in overall, variation (d) resulted in better final objective function’s value and lower number of evaluated solutions than the others.

We then examined the way the path is formed from the initial solution to the guiding solution. In the basic algorithm, the step-size we use to move from an initial solution to intermediate solutions is fixed (pathSqrSize). We examined if using a variable step-size would improve performance. Moving along the path such that the movement starts faster near the initial solution and it becomes slower as it gets closer to the guiding solution, which intensifies the search in the area of the initial solution. This strategy is applied on both directions: from an initial solution to a guiding solution and vice versa. This variation introduces two new parameters to our path relinking procedure: number of iterations required to update the step-size (accelerationPeriod), and the rate of decreasing the step-size (accelerationRate). The net effect is to search more closely to the two known solutions than in the space between them.

We applied both variations to randomly generated connected graph layouts with different number of nodes and edges. The two variations used the same values of all path relinking parameters except for the newly introduced parameters as they are only related to the variable step-size strategy. We ran both of them until they reach the stopping criteria. The results showed that using a variable step-size to move along a path can produce graph layouts with better objective function’s values compared to a fixed step-size strategy. Hence we apply this variable step-size in our algorithm.

## 4. Experimental results

Our research questions were: “Does our tabu search drawing method perform better than hill climbing and simulated annealing approaches?” and “Does coupling the method with path relinking improve the performance of the tabu search graph drawing method?” To answer these questions, we implemented and evaluated the methods. We also implemented hill climbing and simulated annealing, the two commonly used alternative neighbourhood search based approaches for graph drawing.

Three types of evaluations were carried out:

1. finding the best layout that can be achieved (i.e. minimizing the value of the cost function);
2. how long it took to draw a graph to a particular level of quality;
3. how good the quality of the graph was after a fixed optimization time (number of evaluated solutions).

These allow us to examine different possible use cases for graph layout: firstly, generating the best possible layout; secondly, speed to draw an acceptable layout; and thirdly, how good the graph layout can be if there is a fixed time available to produce it.

We first compared the performance of our tabu search method to hill climbing and simulated annealing. As both tabu search and simulated annealing showed a better performance than hill climbing, we then discarded hill climbing for future tests and moved on to comparing our improved tabu search method that has the addition of path relinking against just simulated annealing. Note that, in these experiments, we used the same search space structure, as described previously in section 3 (Fig 1), for all the graph drawing methods.

### 4.1. Parameters tuning

Each method has a number of parameters. Changing the values of those parameters can affect the performance of the method and the quality of the layouts generated by the drawing method. Several experiments were conducted to calibrate the parameters of each method. The experiments show the effect of increasing and decreasing the values of the parameters on the performance of the method and the quality of the layout.

Parameters tuning is a common practice in search based methods. Typically, one parameter is tuned at a time, which might cause suboptimal choices, as parameters could interact in a complex way. However, simultaneous tuning of more parameters leads to a large amount of experiments. There are drawbacks to parameter tuning based on experimentation which can be summarized as follows [41]:

- Parameters are not independent, but testing all different combinations systematically is practically impossible.
- The process of parameter tuning is time consuming, even if parameters are optimized one by one, regardless of their interactions.
- The selected parameter values are not necessarily optimal, even if the effort made for setting them was significant.

However, in this research, we applied a tuning process similar to those conducted in [2, 11, 42, 43]. We performed a systematic incremental exploratory test on a wide range of values for each individual parameter in order to select a robust set of initial values. Then for each single parameter, we tested values while fixing the rest of the parameters. The value which gave the minimum objective function’s value was selected for that parameter, and then we moved to the next parameter to apply the same procedure.

The following are the selected values of the parameters in our tabu search method:

- maxIterations = 40
- tabuCutOff = 4
- intensifyIterations = 5
- intensifyCutOff = 0.005
- tabuDuration = 5

We also performed similar parameter tuning on hill climbing and simulated annealing approaches. Hill climbing algorithm is affected by two parameters: the initial value of the square size used to determine the neighbourhood solutions (initialSquareSize) and the value used to reduce the size of the square (squareReduction). The performance of simulated annealing drawing algorithm is influenced by four parameters: number of iterations for running the algorithm (maxIterations), number of iterations at each temperature (iterPerTemp), the initial temperature used in the annealing process (initialTemp), and the temperature cooling down factor (coolDown). The following are the selected values of the parameters for hill climbing and simulated annealing:

➢ Hill Climbing Parameters
  - initialSquareSize = 512
  - squareReduction = 4

➢ Simulated Annealing Parameters
  - maxIterations = 45
  - iterPerTemp = 15
  - initialTemp = 0.75
  - coolDown = 0.8

All implementations were in Java (version 1.7.0; Java HotSpot™ 64-Bit Server VM 21.0-b17 on Windows 7). The experiments were performed using Lenovo Thinkpad T430, Intel® Core™ i7-3520M, 2.90 GHz and 8 GB RAM.

There are six parameters which affect our improved path relinking procedure: number of iterations to repeat the path relinking procedure (*PRmaxIterations*), the size for the maximum number of solutions that can be added to the reference set of path relinking (*refSize*), the maximum length of the path (*pathLength*), the square size where path relinking candidate solutions are located on its border (*pathSqrSize*), number of iterations required to update the size of the square (*accelerationPeriod*), and the rate of decreasing the searching step-size (*accelerationRate*).

For tuning the values of these parameters, we applied our improved graph drawing algorithm on 100 random connected graphs which were divided into five sets such that each set had a different number of nodes and edges as described in Table 1.

**Table 1:** Characteristics of graph datasets used in tuning parameters of our improved TS+PR graph drawing algorithm.

Graph Set	Nodes	Edges	Density	Label
1	50	147	0.120	N50E147
2	100	519	0.105	N100E519
3	150	1117	0.100	N150E1117
4	200	1791	0.090	N200E1791
5	250	2490	0.080	N250E2490

Since the improved procedure is called within our tabu search drawing algorithm, we used the same values of the parameters of tabu search which we got in an earlier tuning experiment. On the other hand, in order to calibrate the values of the parameters of the improved path relinking, we followed the same incremental testing process we performed with all the other methods. In the first stage of tuning, we selected arbitrary values for the parameters which were chosen based on an observation of a quick experiment. The initial values of the parameters were: *PRmaxIterations* = 4, *refSize* = 20, *pathLength* = 10, *pathSqrSize* = 18, *accelerationPeriod* = 9, *accelerationRate* = 0.01. We started with one parameter, tested it thoroughly with different values, and selected the value which draws layouts with the minimum objective function’s value compared to the other values. If the values of the objective function were too close to each other, we would select the values based on the ones which performed the fewest number of evaluated solutions. We fixed that value of the first parameter and we moved to testing another parameter in the same manner, and so forth.

**Fig 3:**
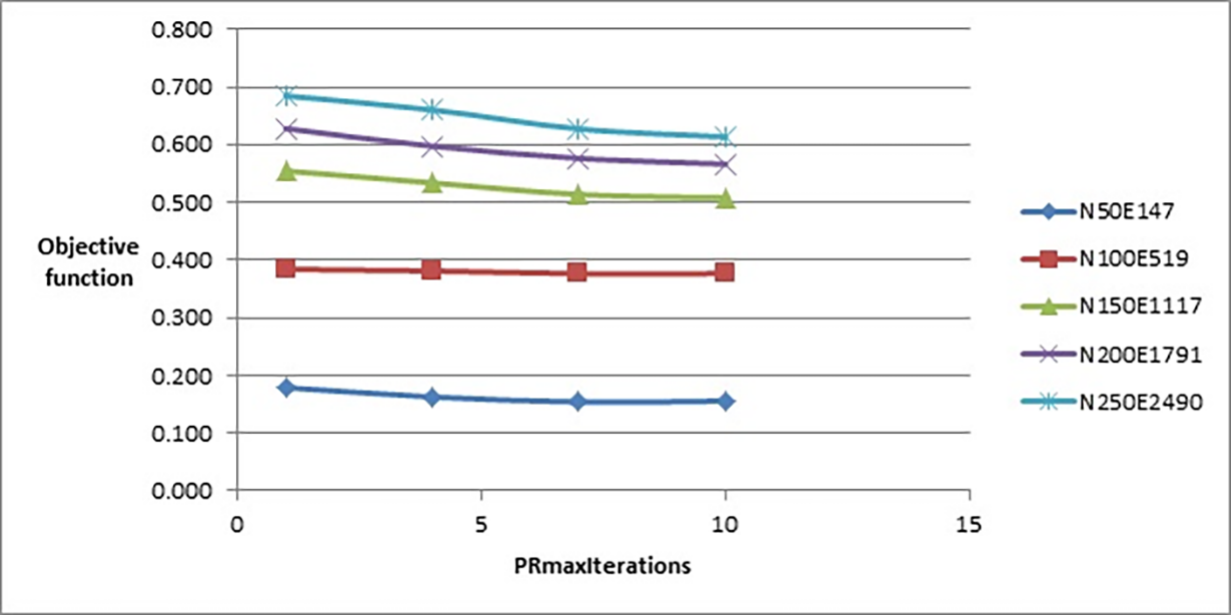
Objective function’s values of the improved drawing algorithm when tuning PRmaxIterations parameter.

We started the tuning process with *PRmaxIterations* parameter by testing the values of the set {1, 4, 7, 10}. Fig 3 shows that increasing the value of this parameter would minimize the value of the objective function of the generated layout. According to the set of values which we tested, the best value to choose was 10.

**Fig 4:**
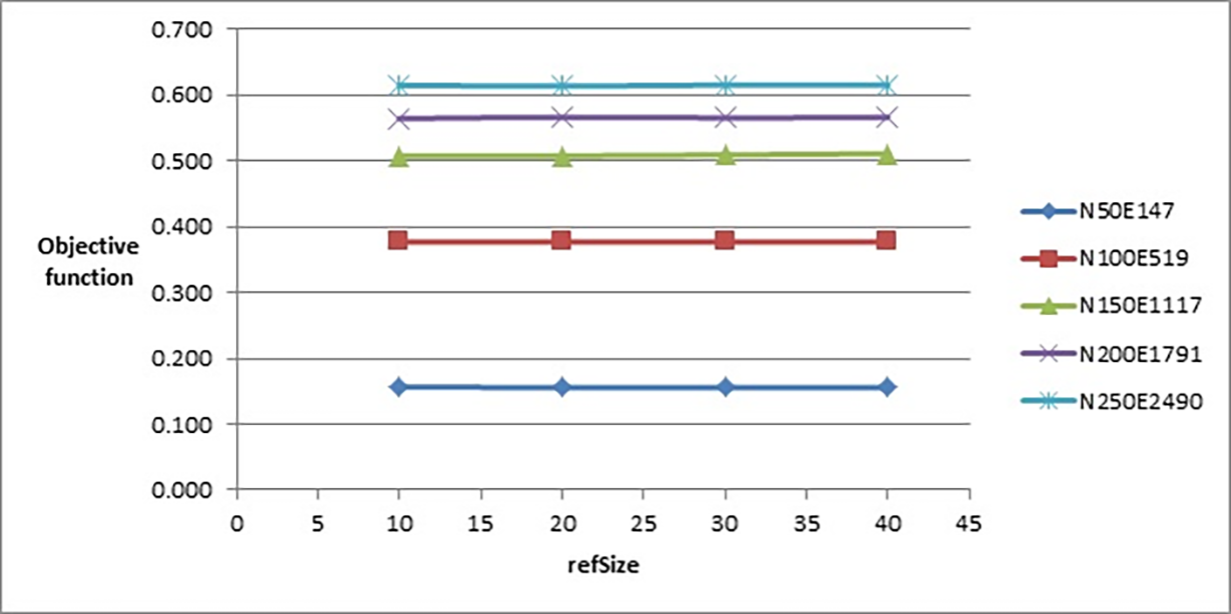
Objective function’s values of the improved drawing algorithm when tuning refSize parameter.

**Fig 5:**
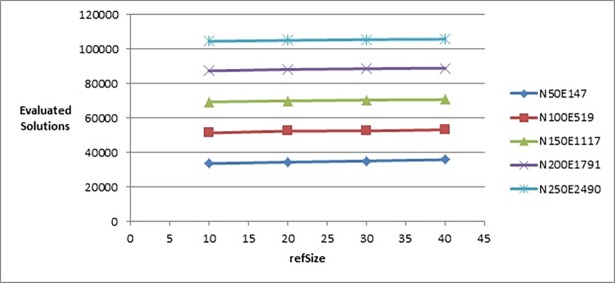
Number of evaluated solutions of the improved drawing algorithm when tuning refSize parameter.

In the next parameter (*refSize*), we selected the set {10, 20, 30, 40} to be used in calibrating this parameter. With reference to Fig 4, the best value for *refSize* that gave the best objective function’s value was 20. Note that, all the tested values led to producing very close values of objective function, but as the value of this parameter increases it slightly increases number of evaluated solutions, as shown in Fig 5. We selected the value 20 as it gave an objective function’s value (on the graphs with label N250E2490) slightly better than the other values and the number of evaluated solutions performed by the algorithm when using this value is less than the evaluated solutions when we test this parameter on the values 30 and 40).

**Fig 6:**
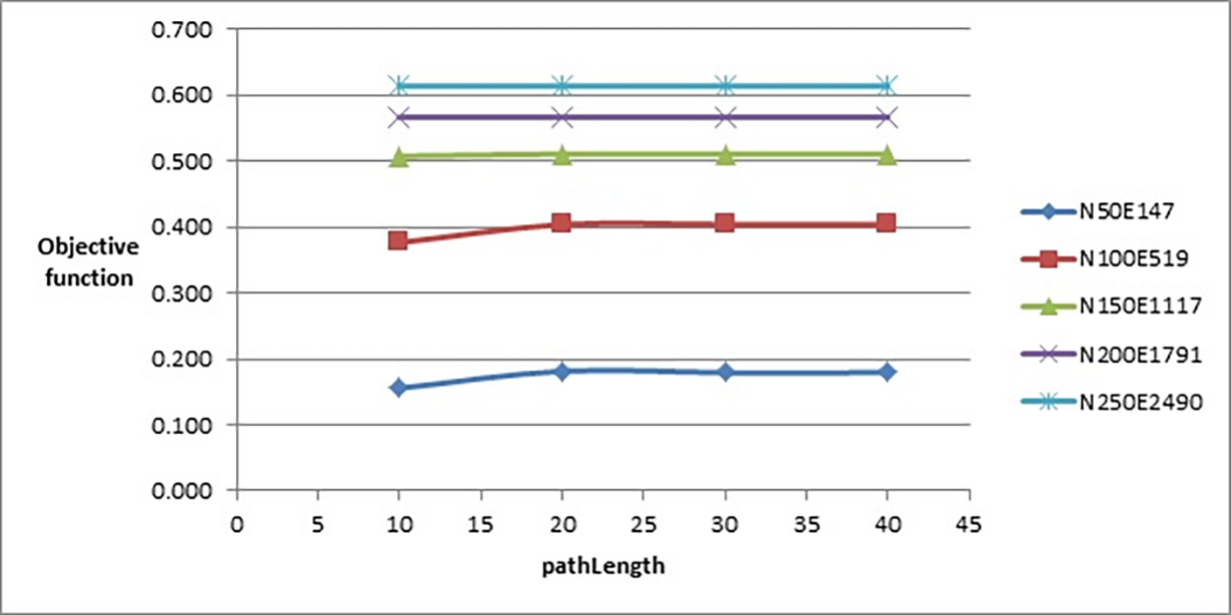
Objective function’s values of the improved drawing algorithm when tuning pathLength parameter.

The length of the path from the initial solution to the target solution (*pathLength*) was tested with the following set of values: {10, 20, 30, 40}. After testing all these values, we selected the value 20. We chose this value although it did not give a better value of objective function compared to the value 10 on small graphs, but it has the same behaviour on larger graphs as shown in Fig 6. We first need to test the effect of initial square size value on longer paths. If the effect is not significant, then we could select the value 10 in further parameters testing.

**Fig 7:**
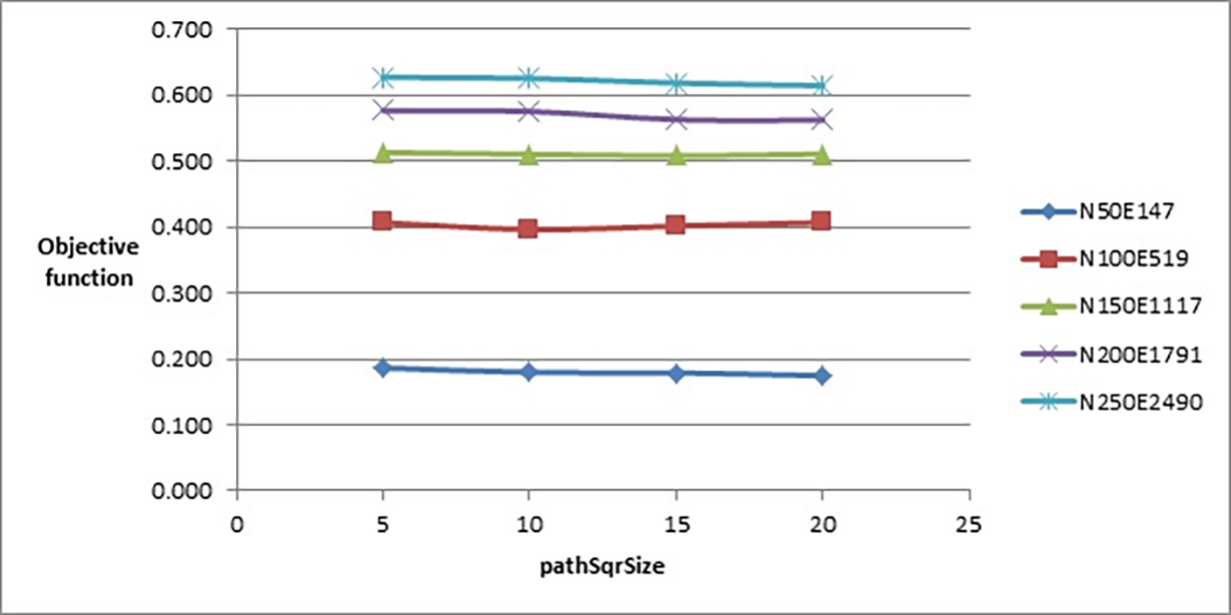
Objective function’s values of the improved drawing algorithm when tuning pathSqrSize parameter.

*pathSqrSize* parameter was tested with the values {5, 10, 15, 20}. According to Fig 7, the best value that could be picked is 20 since the value of the objective function was slightly smaller as the graph size became larger. The value 15 also produced good results but when applied on larger graphs, the value 20 was better.

**Fig 8:**
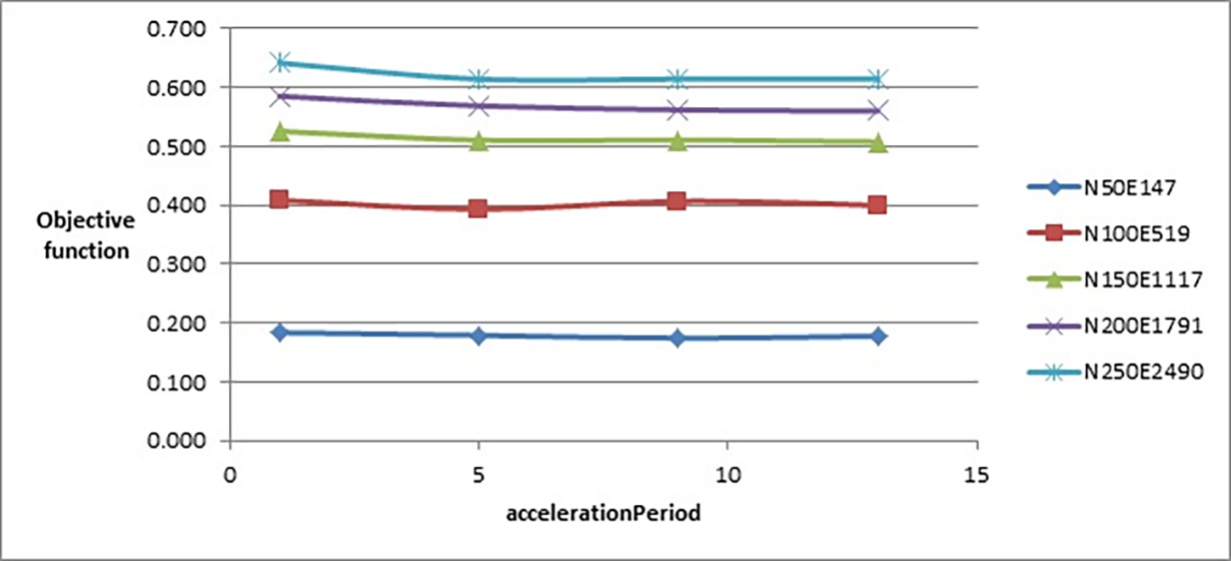
Objective function’s values of the improved drawing algorithm when tuning accelerationPeriod parameter.

**Fig 9:**
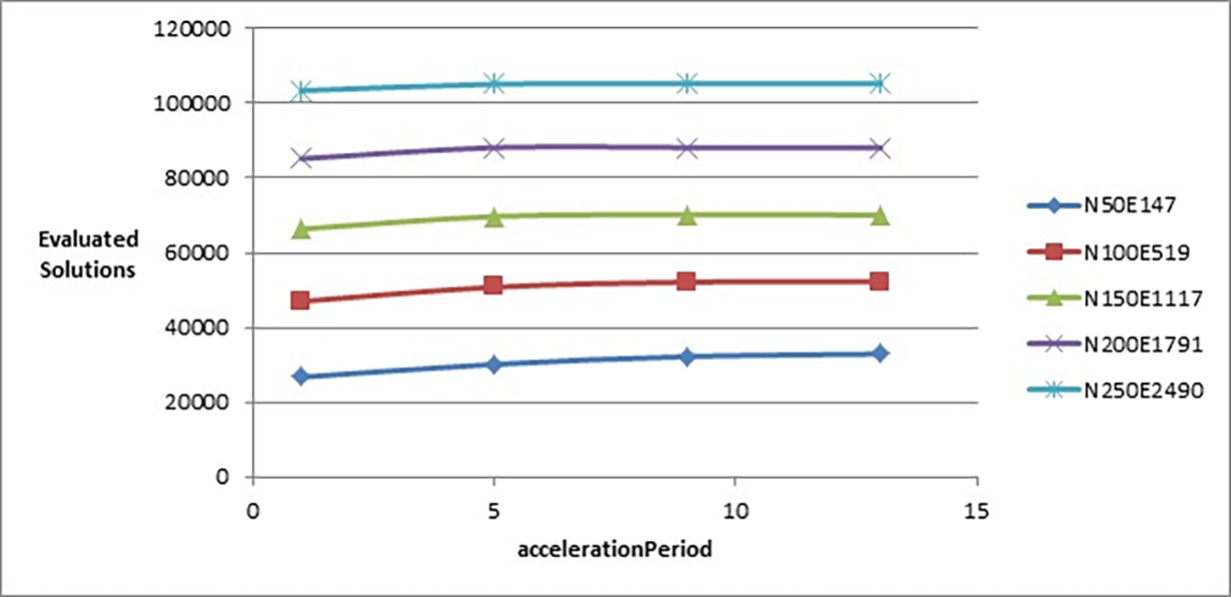
Number of evaluated solutions of the improved drawing algorithm when tuning accelerationPeriod parameter.

To test the effect of *accelerationPeriod* parameter, we tested it with the following values: {1, 5, 9, 13}. Fig 8 shows that changing the value of this parameter did not greatly affect the value of the objective function. But Fig 9 shows that increasing the value of this parameter would slightly increase the number of evaluated solutions. That is why we chose the value 5 although there was no big difference with the objective function’s values produced when *accelerationPeriod* was set to 9 or 13, but it was better on larger graphs with a fewer number of evaluated solutions.

**Fig 10:**
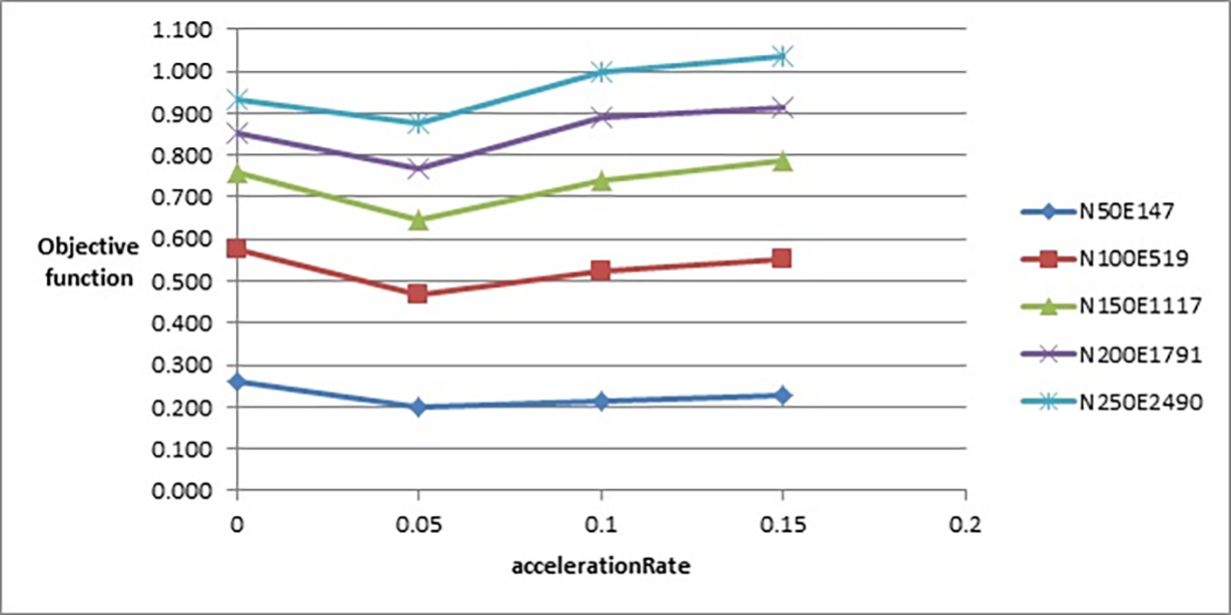
Objective function’s values of the improved drawing algorithm when tuning accelerationRate parameter.

The last parameter, *accelerationRate*, was tested with the values {0, 0.05, 0.1, 0.15}. Increasing the value of this parameter had increased the value of the objective function as shown in Fig 10 when the values went beyond the value 0.05. On the other hand, setting the value 0 to this parameter had produced larger values of objective function compared to those when the value 0.05 was assigned to this parameter. Therefore, we chose the value 0.05 in this stage, but in further experiments, we tested the value of this parameter with a set of values in the range between 0 and 0.05 to examine the behaviour of the objective function in that specific range.

We continued the tuning process by performing another two rounds of tuning similar to the process we followed in the first stage. The first consecutive round examined the effect of the values of the parameters on the value of the objective function when the method performs a fixed number of evaluated solutions, whereas, the second round examined the effect of the values of the parameters on the number of evaluated solutions when the method executes to reach a fixed objective function’s value. In each round, all the parameters started with the values which were chosen in the preceding round. When a parameter is tested, a set of values, which are close to the value that was chosen in the previous round, is selected. After all, the chosen values of the parameters in our path relinking procedure are:

- PRmaxIterations = 4
- refSize = 20
- pathSqrSize = 20
- pathLength = 15
- accelerationPeriod = 7
- accelerationRate = 0.002.

### 4.2. Tabu search versus hill climbing and simulated annealing

First, in order to compare tabu search with hill climbing and simulated annealing, we generated random graph datasets in two categories. The graphs of the first category have the same number of nodes but with different densities (i.e. different number of edges), whereas the graphs of the second category have different number of nodes and edges. The random graph generator is based on the Erdős–Rényi model [44]. The parameters to the random graph generator were the number of nodes and the density of the graph. Random locations for the nodes were generated based on the size of the window where the graph is displayed. Then, the generator chose random nodes as end points of edges. A similar process was performed in [26]. Self-sourcing edges and multiple edges between the same pair of nodes were not allowed. Finally, the graphs generator tested the connectivity of the generated graphs. Only connected graphs were accepted.

There were 80 random graphs in the first category split into 4 groups of 20 test cases. All the graphs in this category had 150 nodes. Each group had a differing number of edges so that the density varied. The graphs in each group had the same number of nodes and edges. See Table 2 for characteristics of the graphs in the first category.

**Table 2:** Characteristics of the graphs in the 1^st^ category used in comparing TS, HC, and SA.

Graph Set	Nodes	Edges	Density
1A	150	558	0.05
2A	150	1117	0.1
3A	150	1676	0.15
4A	150	2235	0.2

The second category also had 80 random graphs, again split into 4 groups. The number of nodes for a group varied, increasing in steps of 50. The value of the density was chosen for each group to avoid too dense graphs. A similar random process used to generate graphs in the first category was applied to this category. See Table 3 for characteristics of the graphs in the second category.

**Table 3:** Characteristics of the graphs in the 2^nd^ category used in comparing TS, HC, and SA.

Graph Set	Nodes	Edges	Density
1B	50	159	0.13
2B	100	569	0.115
3B	150	1173	0.105
4B	200	1990	0.1

The initial layout of nodes for each graph was random. We first applied our tabu search based approach along with hill climbing and simulated annealing approaches to the graphs. Tabu search, path relinking, and hill climbing approaches are deterministic methods which are not influenced by chance. On the other hand, simulated annealing is a stochastic method in which it includes an element of randomness in the neighbourhood searching process. Therefore, this approach has been tested on each individual graph for 30 different runs. Then we find the median of the results for the 30 different runs to compare with the results of tabu search and hill climbing approaches. Note that, we modelled the neighbourhood transition probability of simulated annealing by that described in [2].

To make a comprehensive comparison between the methods, we divided our experiment into three phases. In phase I, we applied the methods on the graphs of the two categories. The methods executed on the 20 test cases in each group of the two categories, and then the average objective function’s value and the average number of evaluated solutions were computed for each group in each method. In this phase, the hill climbing approach executed until it found the best solution that can be reached (i.e. a solution that cannot be further improved). Whereas, simulated annealing and tabu search are more flexible in how they reach a good solution, and hence we ran them using the values of the parameters discussed earlier in the previous subsection.

**Fig 11:**
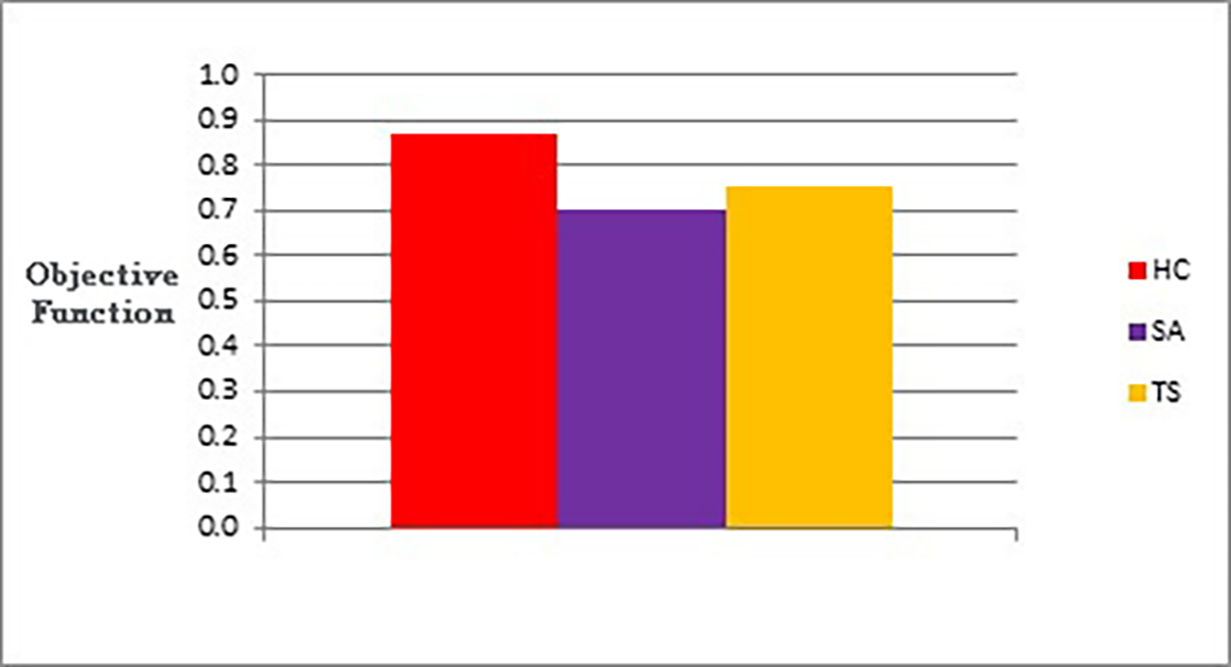
Bar chart of the average overall objective function obtained by TS, HC, and SA when applied on the graphs of the two categories (phase I).

**Fig 12:**
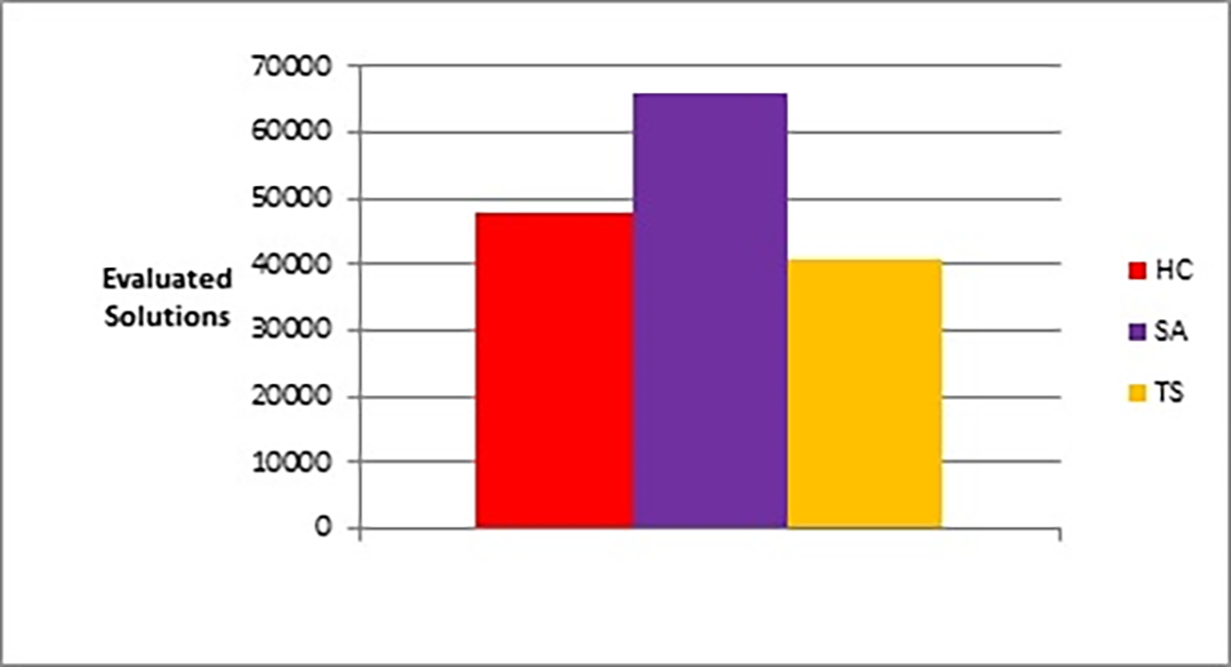
Bar chart of the average overall number of evaluated solutions obtained by TS, HC, and SA when applied on the graphs of the two categories (phase I).

The following figures show bar charts of the results obtained from phase I. Here we are looking for graph layouts with the minimum objective function’s values that can be achieved. Fig 11 clearly shows the difference between the three methods in terms of the lowest objective function’s value that can be obtained. Fig 12 shows the number of evaluated solutions required to achieve this objective function’s value.

In phase II, we investigated the performance of the approaches rather than the quality of the produced layouts. The following process was performed to test which method has the fewest number of evaluated solutions to reach similar values for the objective function:

a We ran the hill climbing method on the graphs until no improvements could be made on the value of the objective function. We started with hill climbing because, in phase I, it produced graph layouts with the worst quality compared to the other two methods. Therefore, simulated annealing and tabu search could typically produce graph layouts with as good quality as the one produced by hill climbing.
b We ran simulated annealing and tabu search methods until they reached an equal or better objective function’s value compared to the one found by the hill climbing drawing algorithm.
c We measured the number of evaluated solutions for each method.

**Fig 13:**
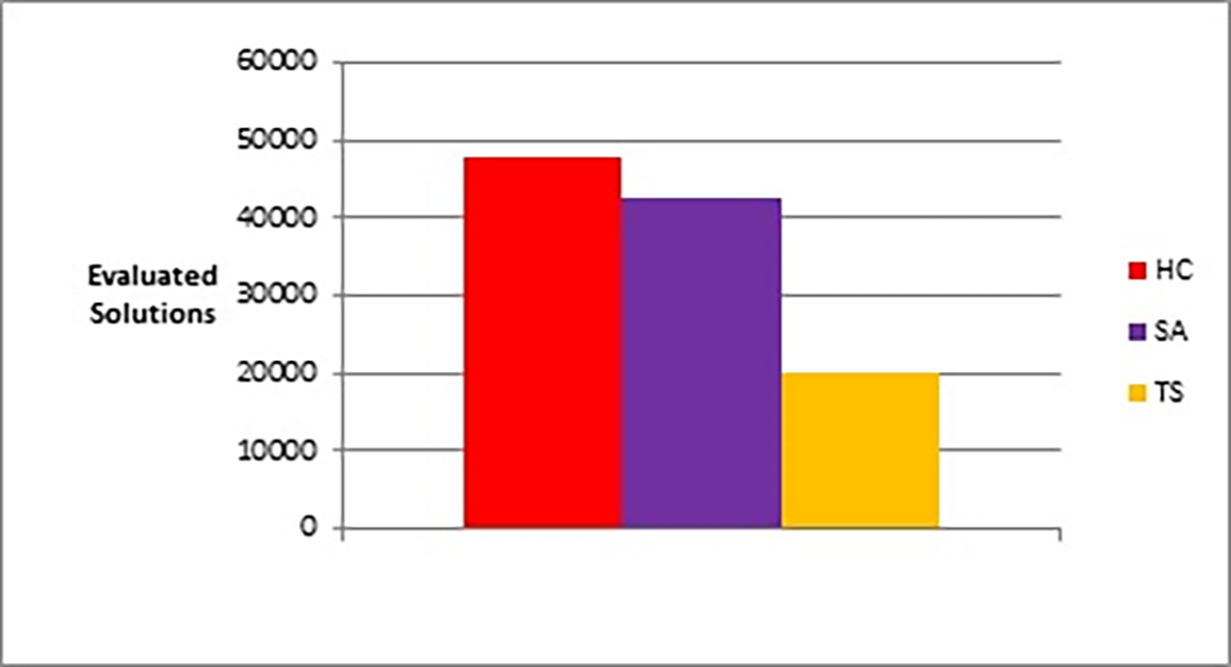
Bar chart of the average overall number of evaluated solutions for TS, HC, and SA when applied on the graphs of the two categories (phase II).

Fig 13 and Table 4 give the results obtained from phase II on the two categories of graphs (refer to the end of subsection 4.3 and section 6 for a complete description and interpretation of the p-value column).

**Table 4:** Statistical analysis of the average overall number of evaluated solutions for TS, HC, and SA when applied on the graphs of the two categories (phase II).

	Evaluated Solutions															
	HC					SA					TS					
Graph Set	Mean	SD	Median	Max	Min	Mean	SD	Median	Max	Min	Mean	SD	Median	Max	Min	p-value
1A	49867	4510	50070	56715	40577	49929	6510	50073	70029	37054	21468	3987	23010	28765	12633	2.51E-07
2A	50622	5004	50131	60846	39727	46822	7606	46122	67079	32120	20851	7054	23205	29366	2272	8.76E-08
3A	53516	7000	52570	65036	42458	46478	7178	47600	60463	32053	25007	8132	27351	41053	6086	2.64E-08
4A	51837	7330	51640	68429	39193	45321	7570	45549	61512	32090	21450	9229	25254	29789	2299	8.76E-08
1B	14523	1939	14205	18779	11801	13136	2727	12388	19220	7822	6665	1953	6142	11819	2690	7.16E-07
2B	32643	4625	32661	44387	25746	27602	6689	26903	41822	16976	12009	5305	11855	22726	2677	5.06E-08
3B	54127	7462	51863	71345	42643	48811	6341	49601	58243	31749	23266	8118.8	23461	44243	5984	4.80E-07
4B	76351	9614	76891	93479	58574	63208	10220	63516	87893	41797	28551	10777	30759	39755	1790	2.64E-08
**Overall**	**47936**	**5935**	**47504**	**59877**	**37589**	**42663**	**6855**	**42719**	**58282**	**28957**	**19908**	**6819**	**21379**	**30939**	**4553**	**< 2.2e-16**

In phase III, we investigated the quality of the layout produced by the drawing algorithms in a fixed amount of time. The following process was performed to test which method produces graph layouts with the lowest values of objective function (best quality) when the three methods apply the same number of evaluated solutions:

a We ran the tabu search method on the graphs for a predefined number of iterations (maxIterations = 40). The number of evaluated solutions is computed and saved. We started with tabu search because in phase I, it generated the least number of evaluated solutions.
b We ran hill climbing and simulated annealing methods until they perform the same number of evaluated solutions performed by the tabu search method.
c We measured the value of the objective function produced by the drawing algorithms in each of the above steps.

**Fig 14:**
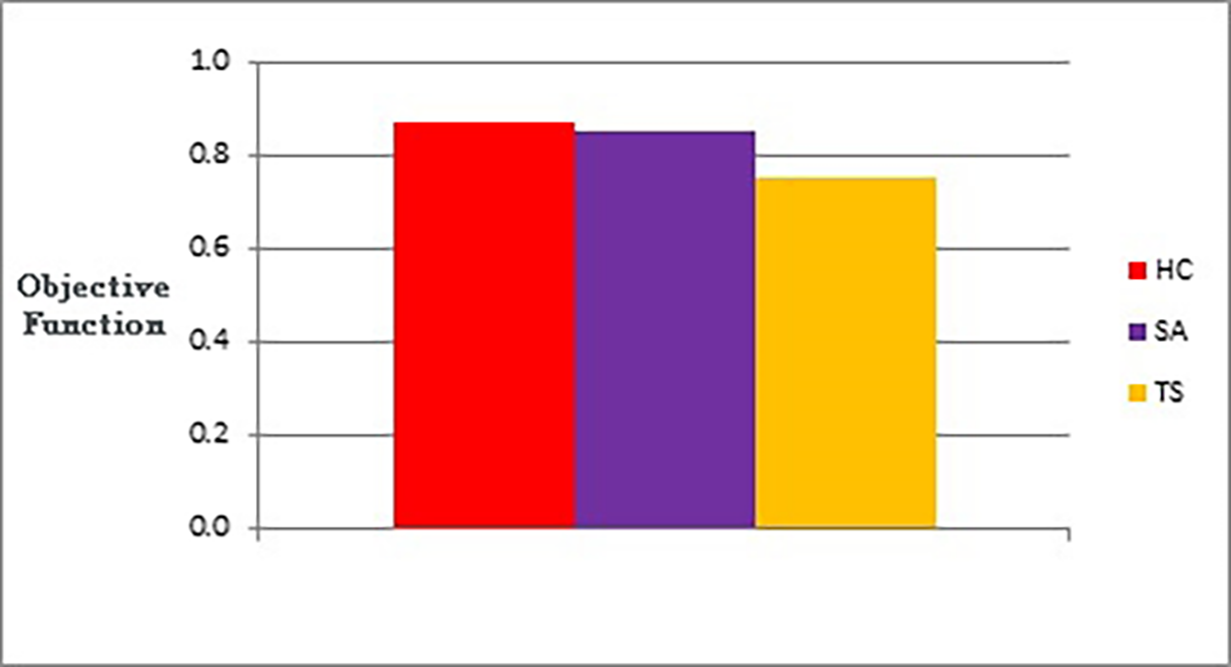
Bar chart of the average overall objective function for TS, HC, and SA when applied on the graphs of the two categories (phase III).

Fig 14 and Table 5 give the results obtained from phase III when applied on the two categories of graphs.

**Table 5:** Statistical analysis of the average overall objective function for TS, HC, and SA when applied on the graphs of the two categories (phase III).

	Objective Function															
	HC					SA					TS					
Graph Set	Mean	SD	Median	Max	Min	Mean	SD	Median	Max	Min	Mean	SD	Median	Max	Min	p-value
1A	0.617	0.063	0.609	0.828	0.502	0.658	0.006	0.659	0.668	0.646	0.505	0.031	0.504	0.558	0.421	2.64E-08
2A	0.904	0.113	0.877	1.211	0.792	0.897	0.006	0.897	0.907	0.886	0.791	0.032	0.784	0.869	0.728	1.38E-07
3A	1.028	0.112	0.989	1.309	0.925	1.015	0.008	1.015	1.033	1.002	0.928	0.04	0.922	1.061	0.889	9.66E-07
4A	1.132	0.114	1.098	1.39	0.988	1.101	0.007	1.1	1.123	1.09	1.017	0.042	1.013	1.154	0.944	1.30E-06
1B	0.487	0.103	0.465	0.827	0.361	0.419	0.011	0.421	0.438	0.39	0.354	0.078	0.332	0.618	0.28	9.80E-07
2B	0.803	0.171	0.746	1.21	0.616	0.696	0.007	0.696	0.713	0.683	0.625	0.054	0.612	0.794	0.551	9.66E-07
3B	0.895	0.097	0.872	1.249	0.803	0.908	0.008	0.909	0.921	0.895	0.805	0.043	0.801	0.948	0.73	4.80E-07
4B	1.122	0.117	1.082	1.517	0.987	1.121	0.01	1.123	1.138	1.102	1.001	0.028	0.995	1.072	0.942	1.36E-07
**Overall**	**0.873**	**0.111**	**0.842**	**1.193**	**0.747**	**0.852**	**0.008**	**0.853**	**0.868**	**0.837**	**0.753**	**0.044**	**0.745**	**0.884**	**0.685**	**< 2.2e-16**

### 4.3. Tabu search with path relinking versus simulated annealing

Here, we want to test the effect of adding path relinking to our tabu search algorithm. In this experiment, we exclude hill climbing as the results of the previous experiment (subsection 4.2) showed that hill climbing performed considerably worse than both tabu search and simulated annealing in all phases. In order to avoid overfitting, where the drawing algorithm could be tailored to the dataset used in the first experiment, we generated new random graph datasets in this experiment which are also divided into two categories, using the same procedure we followed for generating random graphs in our previous comparison.

In the first category, we had 80 random graphs split into 4 groups of 20 test cases. All the graphs in this category had 160 nodes, randomly positioned. Each group had a different number of edges so that the density varied. The graphs in each group had same number of nodes and edges but with different random layouts. See Table 6 for the characteristics of the graphs in the first category. The graphs of the second category were generated in the same way of those graphs of category II described in the previous experiment. See Table 7 for the characteristics of the graphs in the second category.

**Table 6:** Characteristics of the graphs in the 1^st^ category used in comparing PR+TS, TS, and SA.

Graph Set	Nodes	Edges	Density
1C	160	572	0.045
2C	160	1208	0.095
3C	160	1844	0.145
4C	160	2480	0.195

**Table 7:** Characteristics of the graphs in the 2^nd^ category used in comparing PR+TS, TS, and SA.

Graph Set	Nodes	Edges	Density
1D	60	221	0.125
2D	110	659	0.110
3D	160	1272	0.100
4D	210	2139	0.0975

We divided our experiment into three phases similar to those in the previous experiment. In the first phase, all the methods ran until they finish execution.

**Fig 15:**
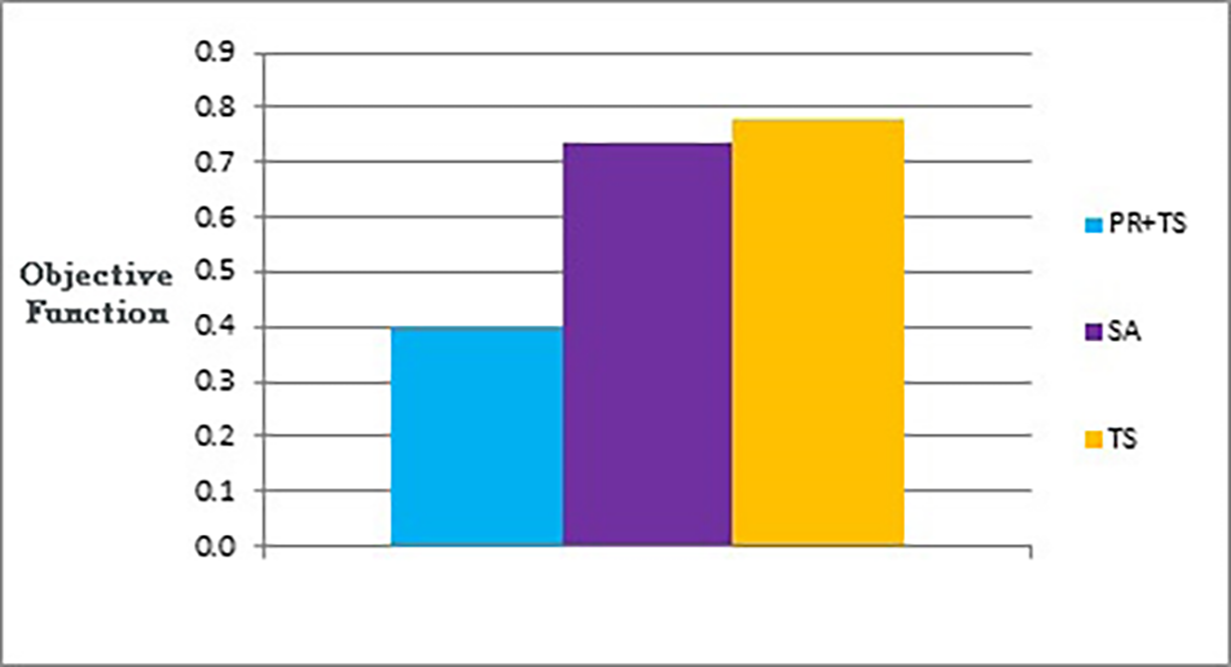
Bar chart of the average overall objective function obtained by PR+TS, TS, and SA when applied on the graphs of the two categories (phase I).

**Fig 16:**
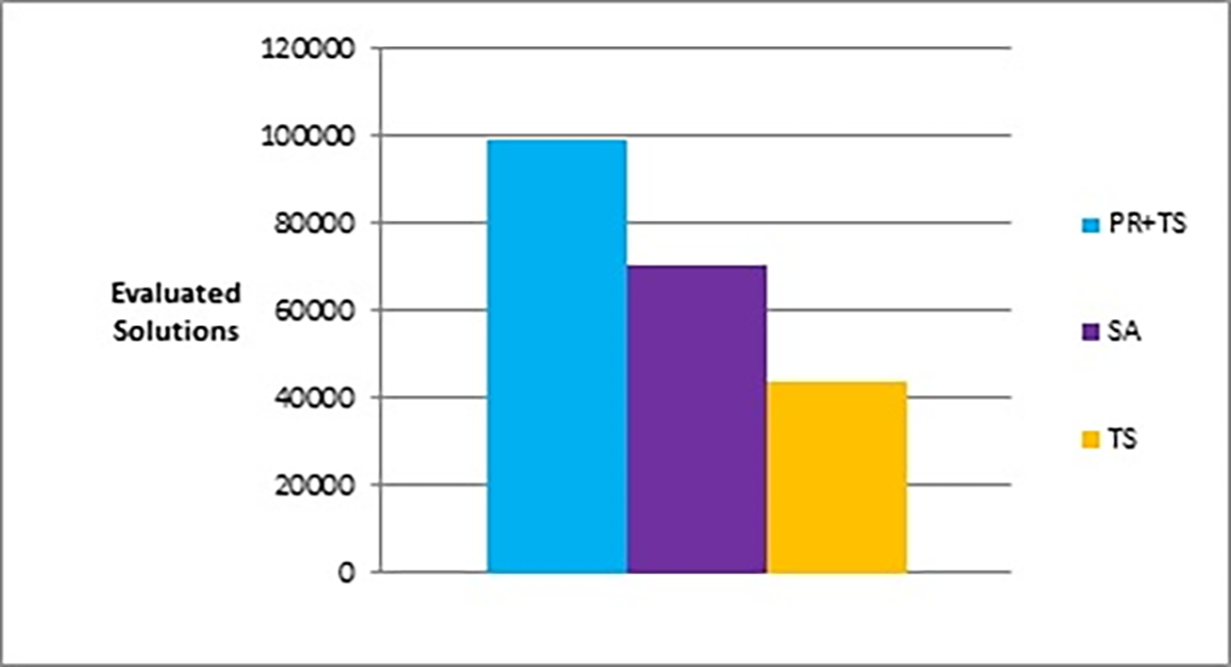
Bar chart of the average number of evaluated solutions obtained by PR+TS, TS, and SA when applied on the graphs of the two categories (phase I).

Figs 15 and 16 show bar charts of the results obtained from phase I, examining how good a layout can the methods achieve. Fig 15 shows the difference between the three methods (the combination of path relinking and tabu search, pure tabu search, and simulated annealing) in terms of the lowest objective function’s value that can obtained. Fig 16, shows the number of evaluated solutions required to reach this objective function’s value.

**Fig 17:**
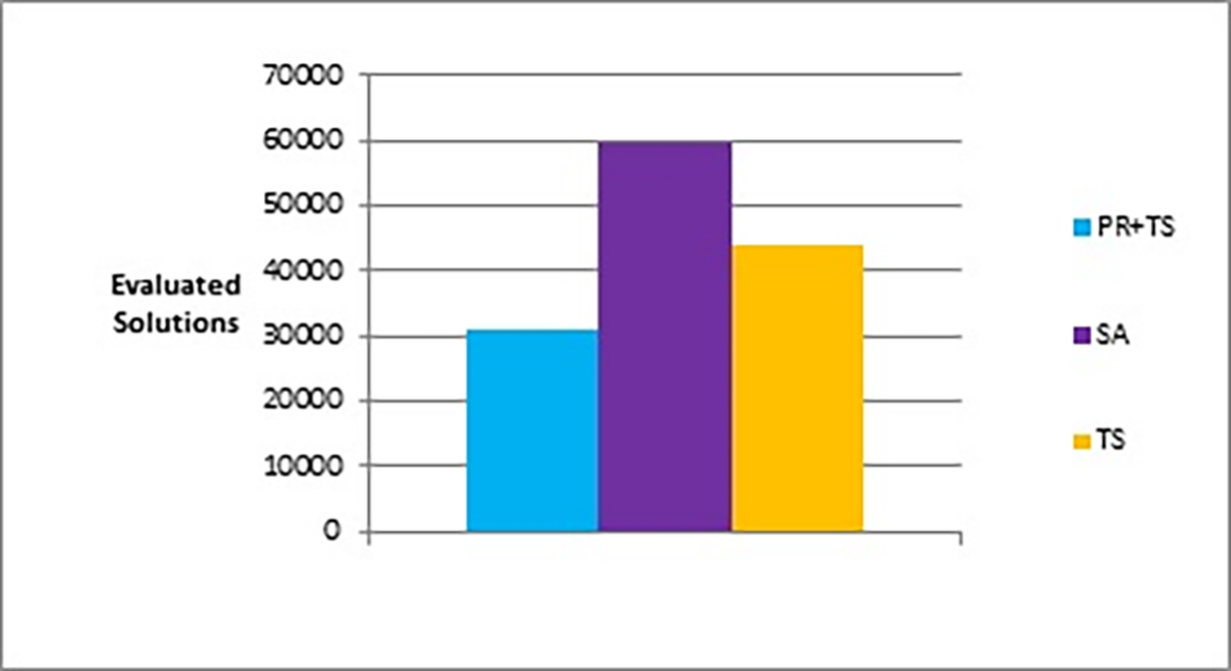
Bar chart of the average overall number of evaluated solutions obtained by PR+TS, TS, and SA when applied on the graphs of the two categories (phase II).

In phase II, where we investigated the performance of the methods, we tested number of evaluated solutions performed by each method to reach similar values for the objective function. We ran tabu search first (as it produced graph layouts with the largest objective function’s values in phase I compared to the others) so that the other methods could easily produce graph layouts with better quality against the ones produced by tabu search. Then, we ran the other methods until they reached an equal or better objective function’s value compared to the one found by the tabu search. Finally, we measured the number of evaluated solutions of each method. Fig 17 and Table 8 show the results obtained from phase II when applied on the two categories of graphs.

**Table 8:** Statistical analysis of the average overall number of evaluated solutions obtained by PR+TS, TS, and SA when applied on the graphs of the two categories (phase II).

	Evaluated Solutions															
	PR+TS					SA					TS					
Graph Set	Mean	SD	Median	Max	Min	Mean	SD	Median	Max	Min	Mean	SD	Median	Max	Min	p-value
1C	39423	6414	36879	55180	29073	72012	3728	73097	76190	65831	47177	171	47181	47501	46872	7.74e-06
2C	32470	8995	30211	56041	14478	62492	6368	62418	76734	48034	47497	266	47499	47867	46776	7.74e-06
3C	32777	7192	29798	44884	21777	62331	6188	60460	74949	54517	47785	172	47786	48061	47555	7.74e-06
4C	31268	6872	29759	52479	23277	62579	5645	62772	76923	53920	47875	194	47936	48180	47550	7.74e-06
1D	17021	3843	17875	21032	8191	25147	4144	26374	29433	13568	17876	163	17902	18170	17572	5.69e-05
2D	30058	6639	30628	38544	17773	46816	4738	48251	53497	35852	32936	183	32880	33314	32680	7.74e-06
3D	29876	7379	29737	44593	8673	61580	9527	61340	76733	37397	47636	308	47675	48023	46603	3.47e-04
4D	32981	7532	29754	50216	21055	85040	10736	83589	99629	68381	62165	294	62119	62739	61676	7.74e-06
**Overall**	**30734**	**6858**	**29330**	**45371**	**18037**	**59750**	**6384**	**59787**	**70511**	**47187**	**43868**	**219**	**43872**	**44232**	**43411**	**< 2.2e-16**

**Fig 18:**
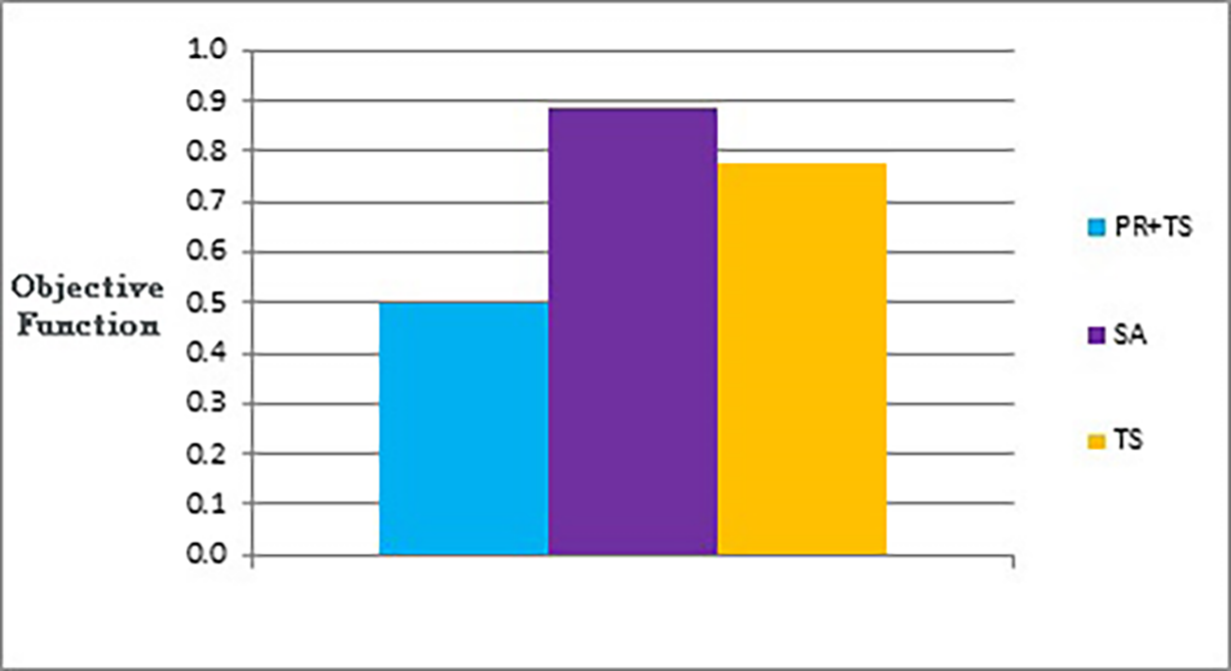
Bar chart of the average overall objective function for PR+TS, TS, and SA when applied on the graphs of the two categories (phase III).

In phase III, we investigated the quality of the layouts produced by the drawing algorithms. We tested which method produced graph layouts with smallest values of objective function when they perform the same number of evaluated solutions. We ran the tabu search method on the graphs for a predefined number of iterations (maxIterations = 40) as described earlier in this subsection. We started with tabu search because in phase I, it generated the least number of evaluated solutions. We ran the other methods until they perform the same number of evaluated solutions performed by the tabu search method. Finally, we measured the value of the objective function produced by each drawing algorithm. Fig 18 and Table 9 show the results obtained from phase III on the two categories of graphs.

**Table 9:** Statistical analysis of the average overall objective function for PR+TS, TS, and SA when applied on the graphs of the two categories (phase III).

	Objective Function															
	PR+TS					SA					TS					
Graph Set	Mean	SD	Median	Max	Min	Mean	SD	Median	Max	Min	Mean	SD	Median	Max	Min	p-value
1C	0.343	0.015	0.347	0.384	0.320	0.664	0.007	0.663	0.676	0.646	0.506	0.024	0.505	0.549	0.463	7.74e-06
2C	0.451	0.014	0.451	0.481	0.419	0.930	0.008	0.930	0.945	0.912	0.825	0.035	0.821	0.923	0.741	7.74e-06
3C	0.531	0.031	0.532	0.595	0.474	1.051	0.007	1.052	1.064	1.038	0.951	0.022	0.956	0.988	0.906	7.74e-06
4C	0.650	0.029	0.656	0.692	0.595	1.139	0.008	1.139	1.152	1.117	1.042	0.020	1.038	1.084	0.988	7.74e-06
1D	0.561	0.048	0.564	0.631	0.440	0.485	0.011	0.486	0.507	0.464	0.398	0.058	0.388	0.591	0.328	5.69e-05
2D	0.407	0.023	0.404	0.458	0.377	0.727	0.009	0.729	0.741	0.709	0.634	0.027	0.628	0.700	0.592	7.74e-06
3D	0.466	0.021	0.469	0.510	0.425	0.942	0.009	0.946	0.959	0.920	0.857	0.076	0.846	1.148	0.782	5.69e-05
4D	0.558	0.029	0.557	0.612	0.493	1.162	0.012	1.167	1.176	1.123	1.021	0.037	1.028	1.080	0.946	7.74e-06
**Overall**	**0.496**	**0.026**	**0.497**	**0.545**	**0.443**	**0.887**	**0.009**	**0.889**	**0.902**	**0.866**	**0.779**	**0.037**	**0.776**	**0.883**	**0.718**	**< 2.2e-16**

To test the effect of randomness in generating the initial graph layouts used in comparing the methods, we performed a statistical significance test on the results generated from the three phases. To demonstrate that there is a statistical significant difference between the methods, we applied the Friedman test [45] which is a non-parametric test for testing the differences between several samples. This test requires no prior knowledge of the distribution of the data.

We ran the methods on 20 randomly generated test cases for each group of graphs in the first and the second categories. Note that, in simulated annealing, we calculated the median of 30 runs for each test case instead of finding the mean (median is more reliable in avoiding outliers) and consequently we got 20 medians (since we find the mean of 30 medians for each test case). Then we compared them with the results of the means computed by the other search based methods using Friedman test with a significance level α = 0.05. See the p-values in the last column of Table 4, Table 5, Table 8, and Table 9.

The Friedman test allowed us to conclude that there is a significant difference between the methods, but it does not show how each method differs from the other. Therefore, a post-hoc test for multiple comparisons between the methods had to be conducted. The Bonferroni method [46, 47] is a simple method that allows pairwise comparisons, see Tables 10 and 11 for the p values. Note that all the statistical tests were conducted using R statistical package i386 (version 3.1.1).

**Table 10:** Bonferroni statistical test (p-values) on number of evaluated solutions of the methods when applied on graph layouts of the two categories (Phase II).

	Evaluated Solutions	
	PR+TS	SA
**SA**	< 2e-16	-
**TS**	2.7e-16	< 2e-16

**Table 11:** Bonferroni statistical test (p-values) on the objective function values of the methods when applied on graph layouts of the two categories (Phase III).

	Objective Function	
	PR+TS	SA
**SA**	< 2e-16	-
**TS**	< 2e-16	2.6e-06

In terms of threats to validity, three deterministic algorithms and one stochastic algorithm were examined. The deterministic methods were applied once on the same initial graph layout whereas the stochastic method was applied 30 times on the same graph. The main internal threat is in the implementation of the algorithms. The methods were implemented by the same coder, and were run on the same machine. There is the possibility that one of the methods was implemented in a more efficient way. However, the methods share substantial code which increases confidence that none was particularly disadvantaged. In addition, a systematic parameter tuning method was applied. In terms of external threats, a threat to the generalizability of the results is possible. Selection bias was avoided by using randomly generated graphs (except in the parameters of the generation algorithm, such as number of nodes and edges). However, randomly generated graphs generally do not have the same characteristics as real world graphs. In section 5, we explore the methods applied to real world standard public datasets sourced from the Internet.

**Fig 19:**
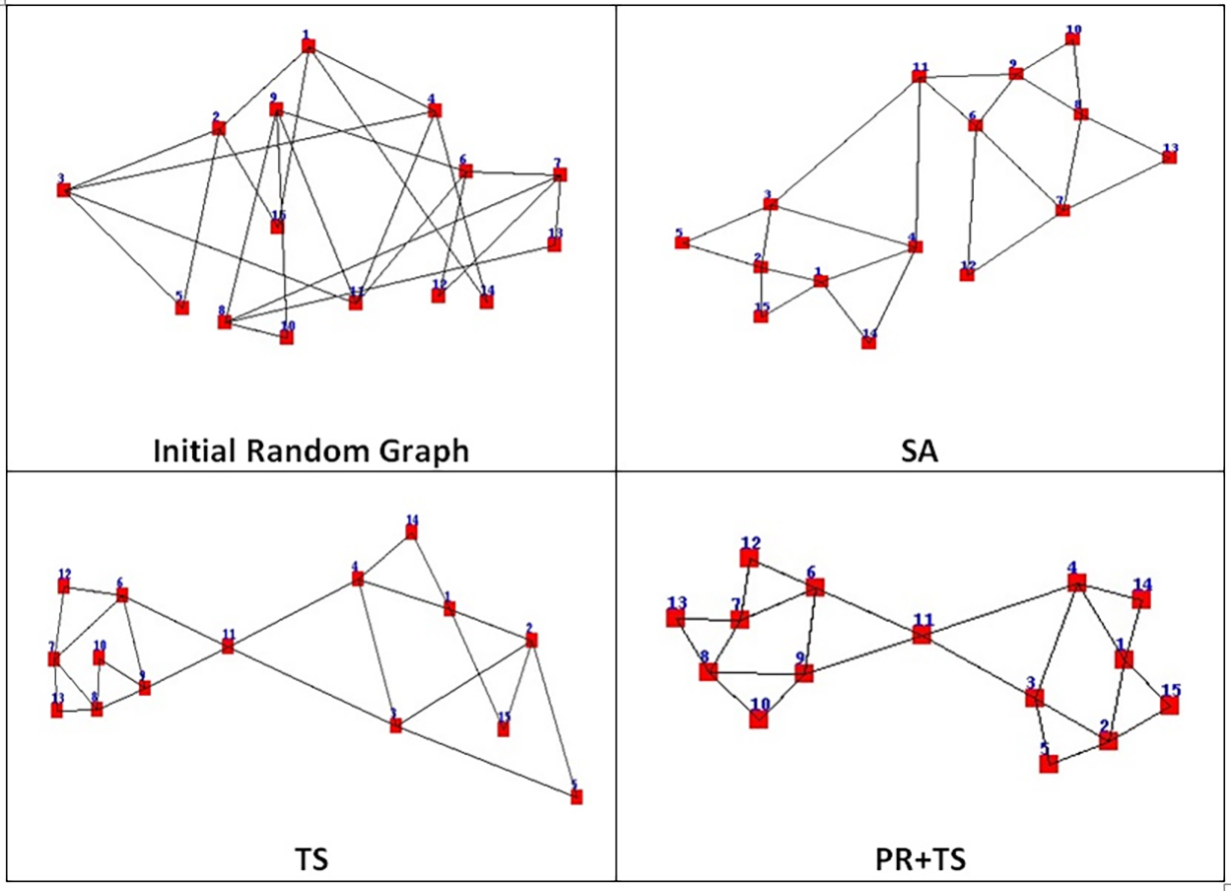
A random graph layout with 15 nodes and 24 edges drawn by SA, TS, and PR+TS in Phase I.

Fig 19 demonstrates a random graph layout with 15 nodes and 24 edges drawn by simulated annealing, tabu search, and our version of path relinking.

### 4.4. Scalability and performance analysis

In order to test the scalability of our method and its ability to work effectively on large graph datasets, we ran our method against simulated annealing on randomly generated large graphs according to phase I as described in subsection 4.2. Note that we excluded hill climbing from this comparison as the statistical tests in subsection 4.2 showed that hill climbing is considerably worse than the other methods. We ran simulated annealing 30 times on each dataset, and the median value was recorded for each set. The graphs were generated using the same generator discussed in subsection 4.2 and in [44]. We started with a graph dataset of 1000 nodes and 3003 edges and we kept increasing the number of nodes and edges as we move from one dataset to another as shown in Table 12. We stopped increasing the size of the datasets when we got a very long execution time for one of the tested methods (almost half a day).

**Table 12:** Characteristics of the graph datasets used in scalability testing.

Graph Set	Nodes	Edges
1	1000	3003
2	1500	4503
3	2000	6003
4	2500	7503
5	3000	9003
6	3500	10503
7	4000	12003
8	4500	13503
9	5000	15002
10	5500	16503

**Fig 20:**
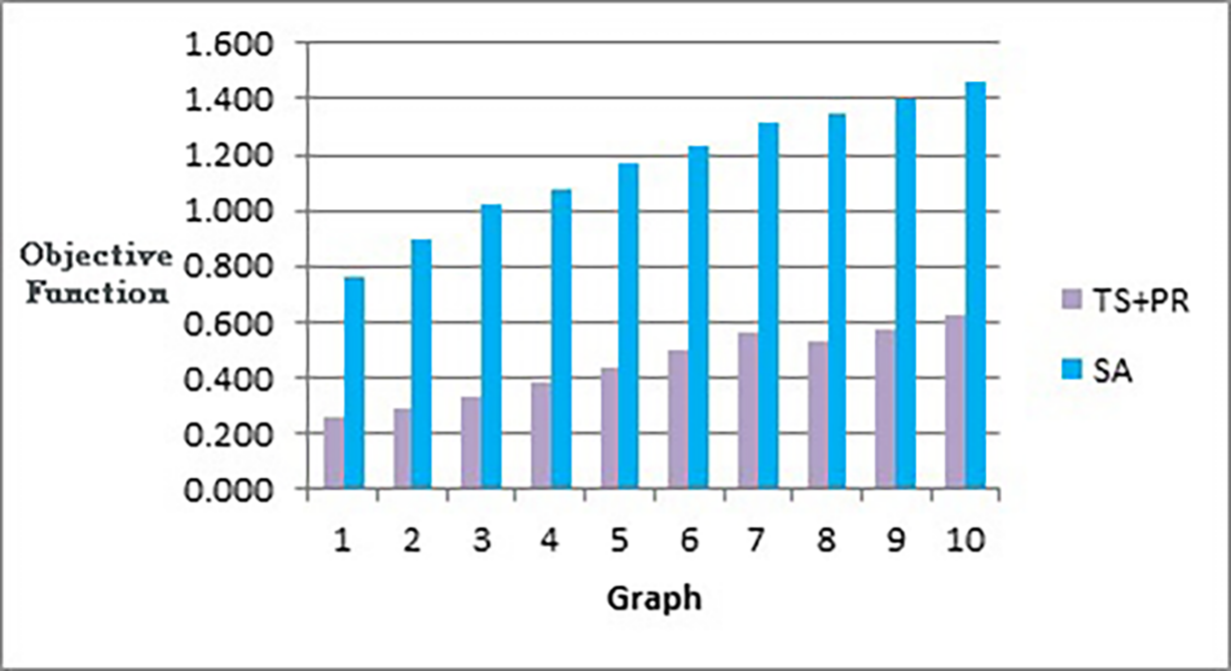
Bar chart of the objective function values obtained by TS+PR and SA when applied on graph datasets in Table 12 (phase I) for scalability testing.

**Fig 21:**
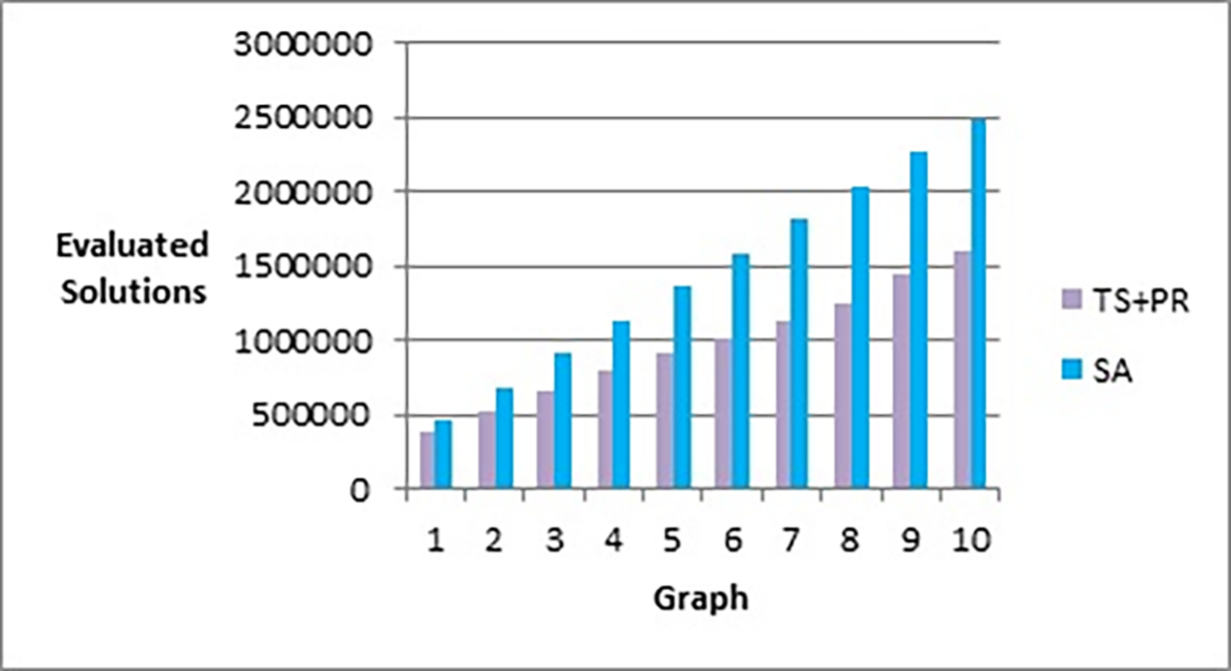
Bar chart of number of evaluated solutions obtained by TS+PR and SA when applied on graph datasets in Table 12 (phase I) for scalability testing.

**Fig 22:**
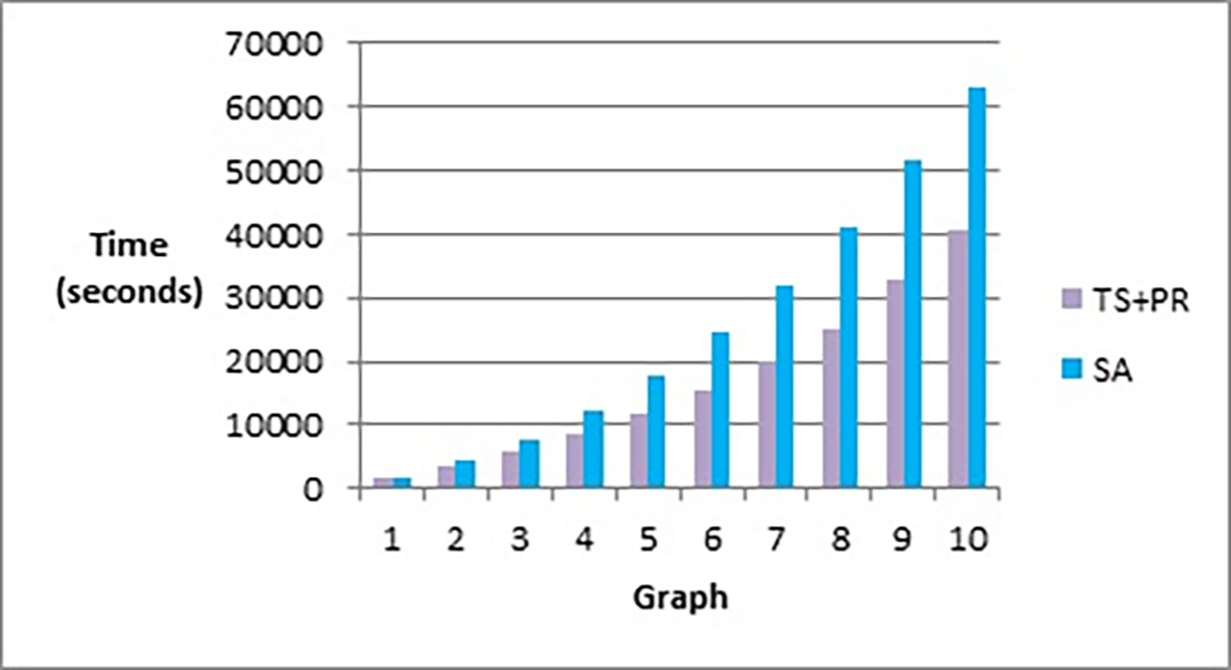
Bar chart of execution time in seconds obtained by TS+PR and SA when applied on graph datasets in Table 12 (phase I) for scalability testing.

Fig 20 shows that our method effectively minimizes the value of the objective function and outperforms simulated annealing regardless of how large the size of the graph is. Also, as Figs 21 and 22 show, the speed of this minimization process is efficient in our method compared to simulated annealing as the graph size increases. The figures show that increasing the number of nodes and edges (i.e. increasing the size of the graph) would increase the number of evaluated solutions and execution time for simulated annealing and our method as well, but with different rates of increase. Note that execution time would be shorter if we test the methods for drawing graph layouts with single criterion. However, since our objective function contains multiple measures, it took a longer time to execute as some measures a have long computation time.

**Fig 23:**
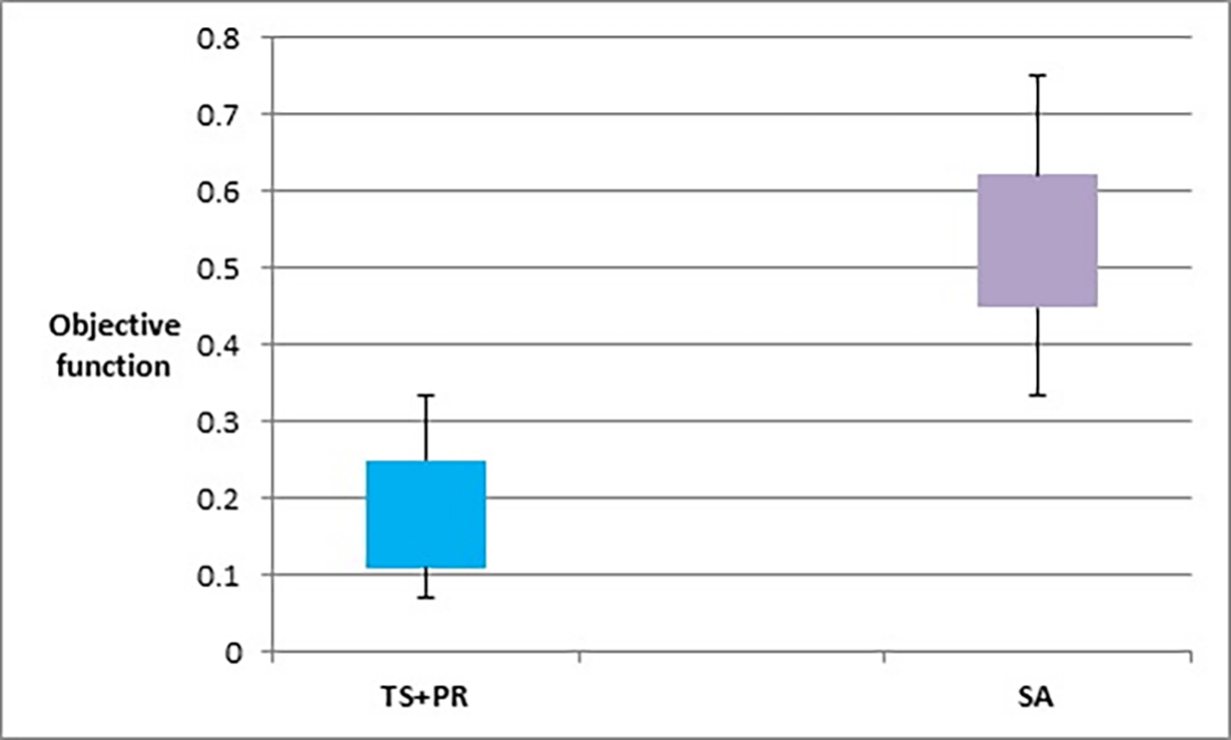
Box plot chart of the overall objective function values obtained by TS+PR and SA when applied on graph datasets with increasing number of nodes and edges (Table 12).

**Fig 24:**
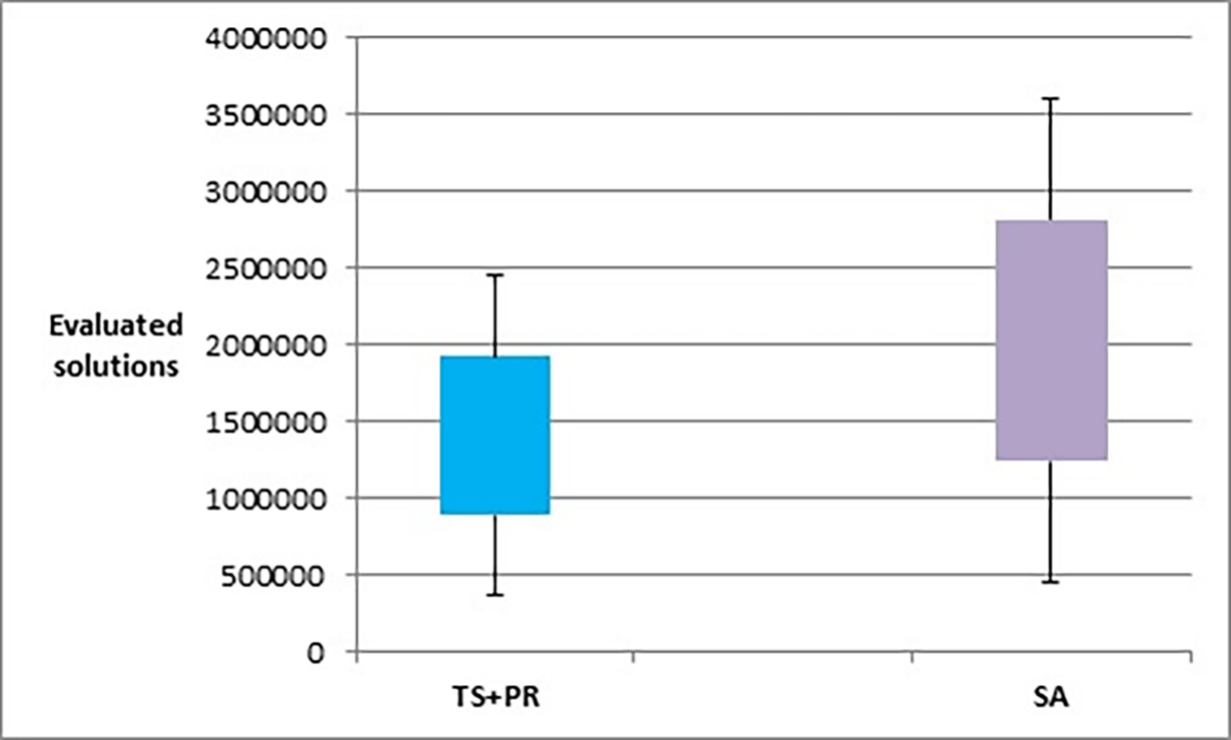
Box plot chart of the overall number of evaluated solutions obtained by TS+PR and SA when applied on graph datasets with increasing number of nodes and edges (Table 12).

**Fig 25:**
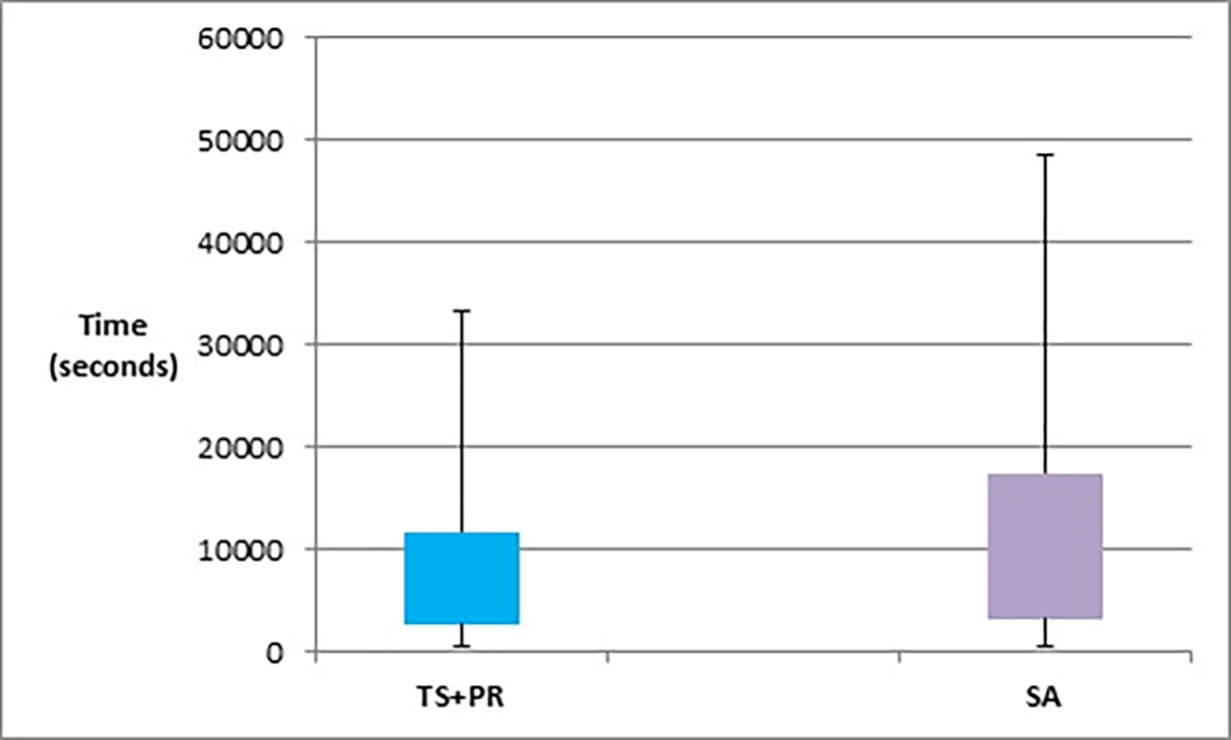
Box plot chart of the overall time in seconds obtained by TS+PR and SA when applied on graph datasets with increasing number of nodes and edges (Table 12).

Figs 23–25, show the overall performance of our method when being applied on a set of graphs with increasing number of nodes and edges, as described in Table 11, in terms of objective function’s values, number of evaluated solutions, and execution time in seconds respectively.

**Fig 26:**
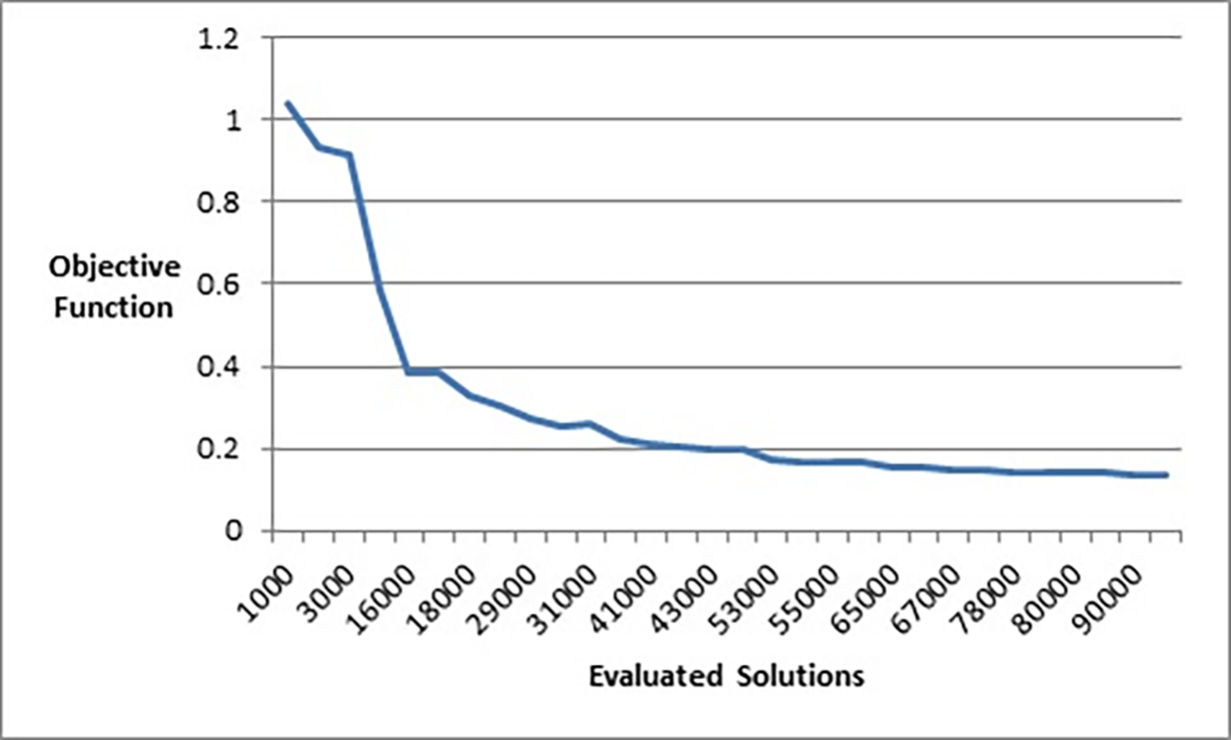
The change of the value of the objective function as the number of evaluated solutions increases.

In order to examine the behaviour of our method on the value of the objective function as the number of evaluated solutions increases, we ran the method on several graphs with the same size of 105 nodes and 441 edges but with different initial layouts. The average value of the objective function was recorded at different points during the execution time of the method. Fig 26 shows the change in the value of the objective function as the number of evaluated solutions increases.

## 5. Real world graph datasets

**Fig 27:**
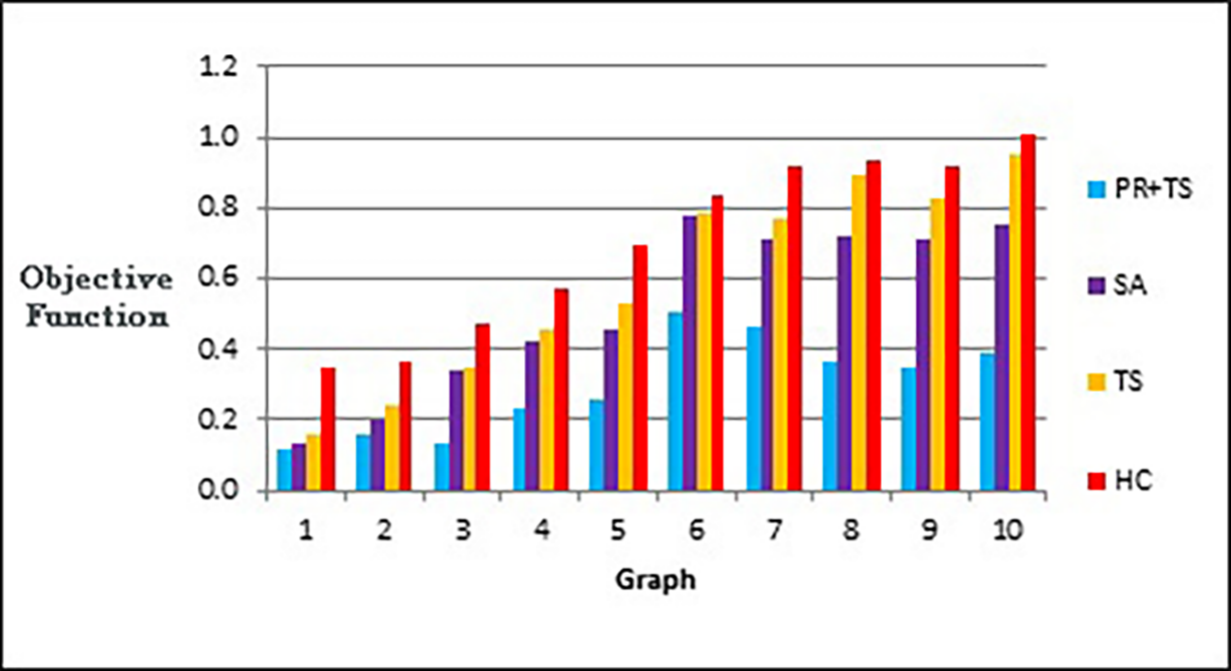
Bar chart of the objective function values obtained by the methods when applied on graph datasets in Table 13 (phase I).

**Fig 28:**
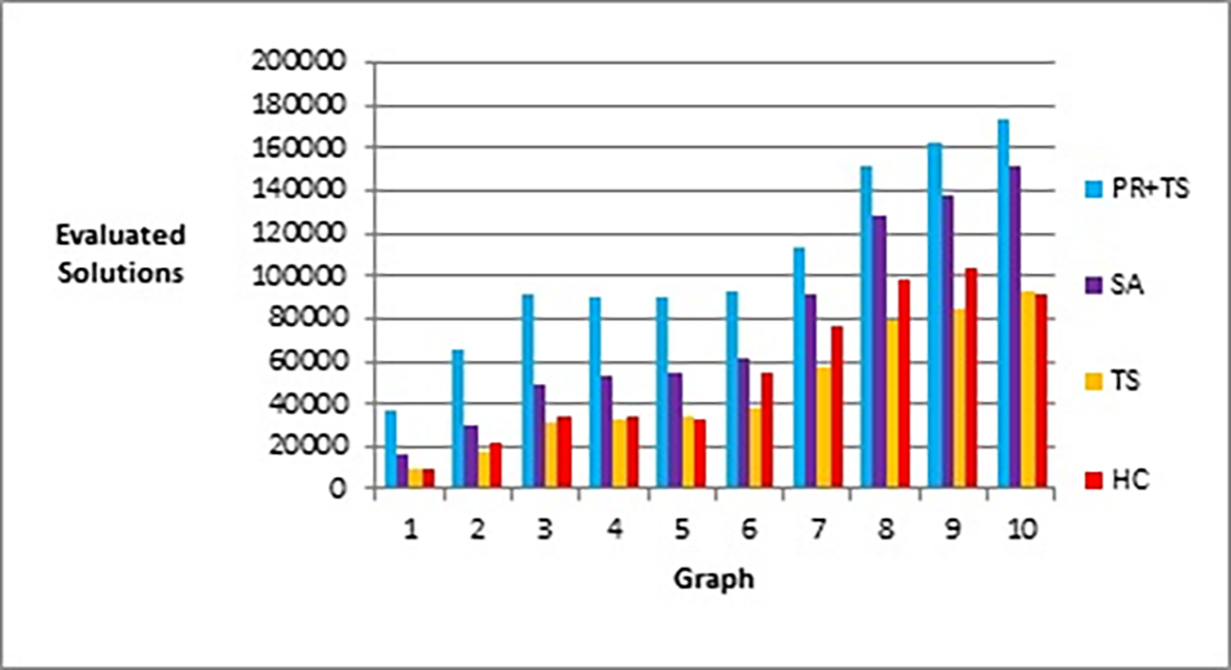
Bar chart of number of evaluated solutions obtained by the methods when applied on graph datasets in Table 13 (phase I).

After performing several experiments on random graphs, we tested our methods on real world graph datasets to demonstrate that we can reproduce similar results in a real world setting. We selected 10 different datasets from different sources as shown in Table 13 that also shows number of nodes, number of edges, and density in each graph. The graphs have different sizes with different densities. The initial layout of the nodes in each graph was generated randomly. Hill climbing, tabu search and path relinking algorithms have run once on the same initial layout whereas simulated annealing has run 30 times, as we did previously, and we calculated the median for each 30 runs which was used in comparison with the results of the other methods. We tested the methods according to phases I, II, and III described in the previous section. The results of the experiments are shown in the following figures. Figs 27 and 28 show the results of applying the methods on real data graphs described in Table 13 according to phase I.

**Table 13:** Real world graph datasets characteristics and sources.

Graph	Nodes	Edges	Density	Source	Description
1	34	78	0.139	[48]	A social network of friendships between 34 members of a karate club at a US university in the 1970s
2	62	159	0.084	[49]	An undirected social network of frequent associations between 62 dolphins in a community living off Doubtful Sound, New Zealand
3	105	441	0.081	[50]	Books about US politics sold by the online bookseller Amazon.com. Edges represent frequent co-purchasing of books by the same buyers, as indicated by the "customers who bought this book also bought these other books" feature on Amazon
4	112	425	0.068	[51]	the network of common adjective and noun adjacencies for the novel "David Copperfield" by Charles Dickens
5	115	613	0.094	[52]	the network of American football games between Division IA colleges during regular season Fall 2000
6	128	2075	0.255	[53]	A network contains the carbon exchanges in the cypress wetlands of South Florida during the wet season
7	198	2742	0.141	[54]	List of edges of the network of Jazz musicians
8	277	1918	0.05	[55]	C. elegans global network of 277 neurons, and the spatial positions of the neurons as two-dimensional coordinates
9	297	2148	0.049	[56]	Neural network of the nematode C. Elegans
10	332	2126	0.039	[57]	Undirected weighted graph for US Air flights

**Fig 29:**
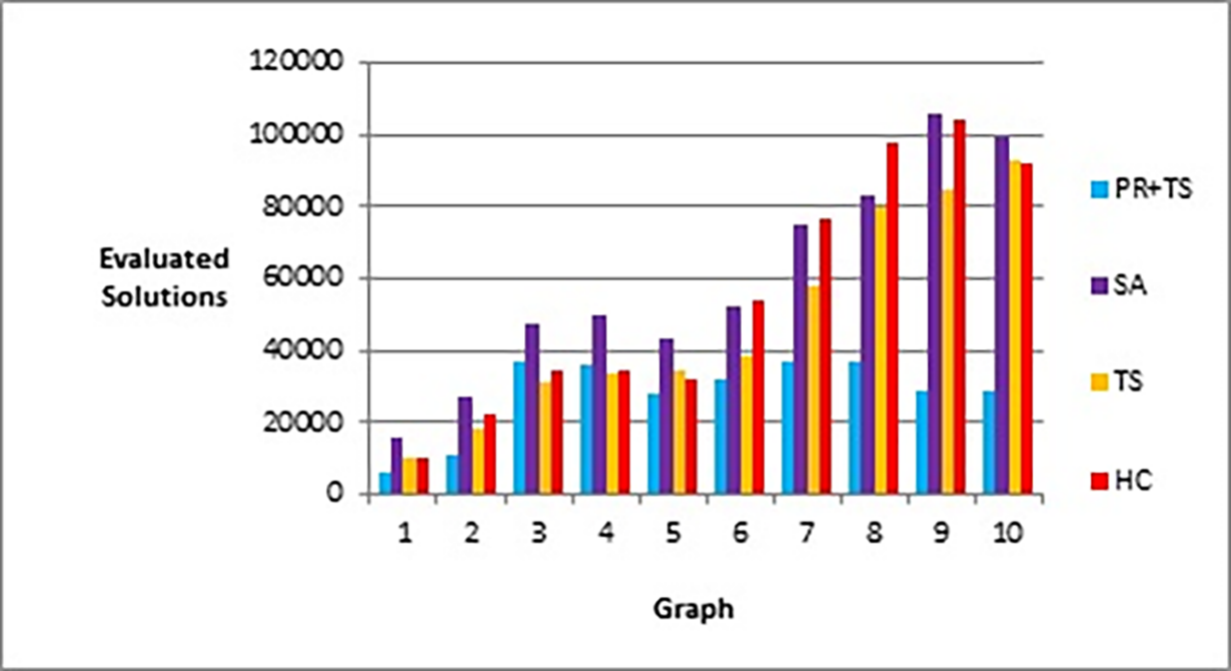
Bar chart of number of evaluated solutions obtained by the methods when applied on graph datasets in Table 13 (phase II).

**Fig 30:**
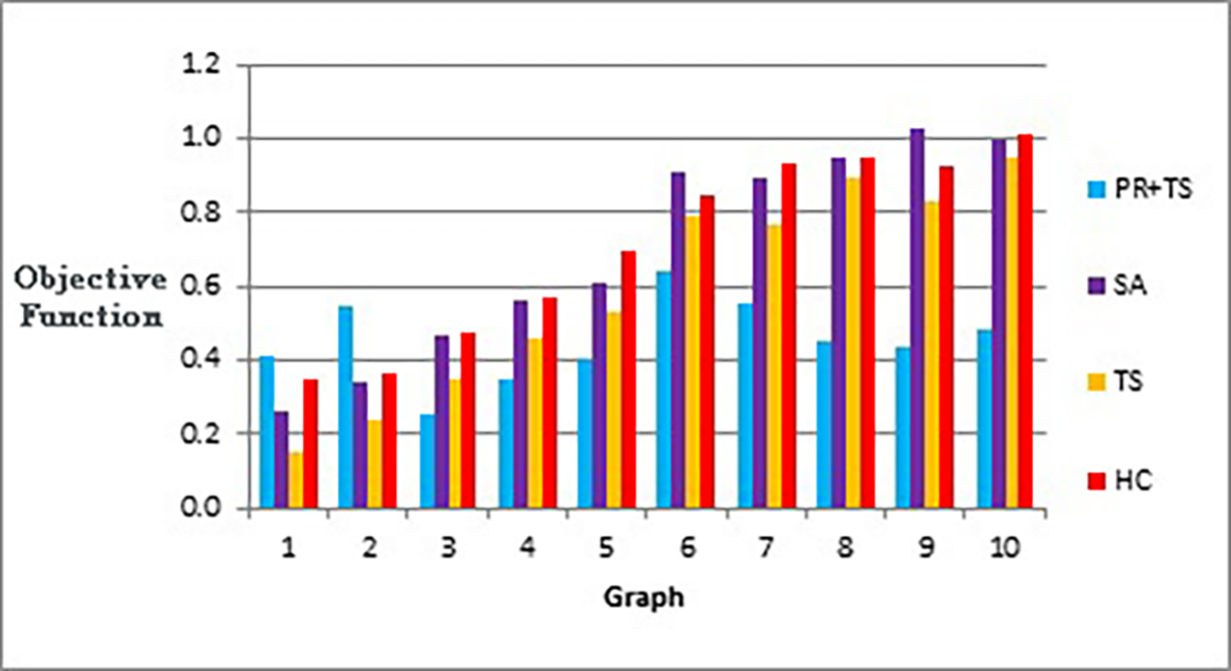
Bar chart of the objective function values obtained by the methods when applied on graph datasets in Table 13 (phase III).

Fig 29 represents number of evaluated solutions performed by each method when testing them on the real world graphs described in Table 13 according to phase II, while Fig 30 demonstrates the values of the objective function produced by each method when they follow the experiment described in phase III when applied on the same set of data.

**Fig 31:**
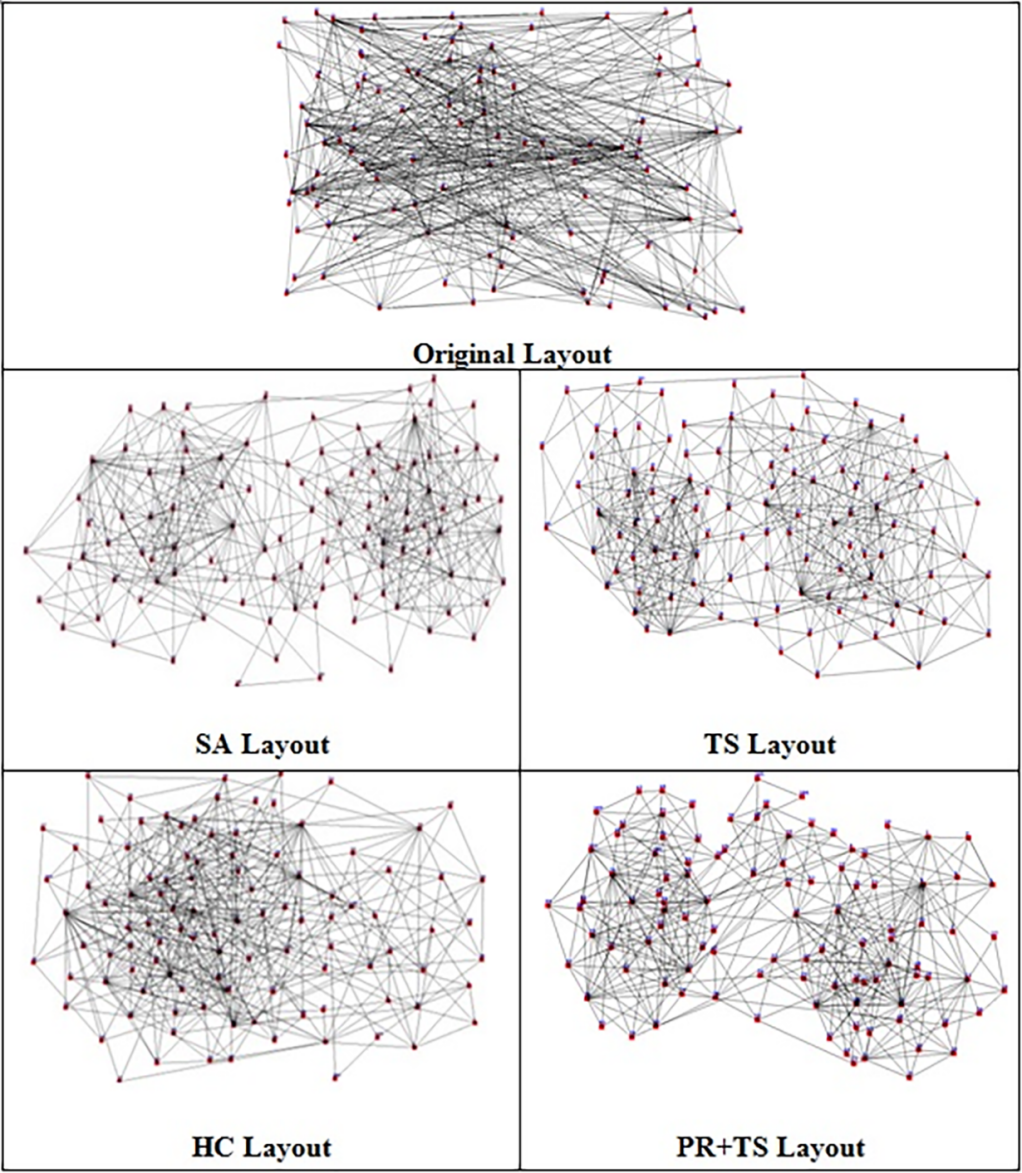
Layouts of graph dataset 3 (listed in Table 13) produced by all the methods.

**Fig 32:**
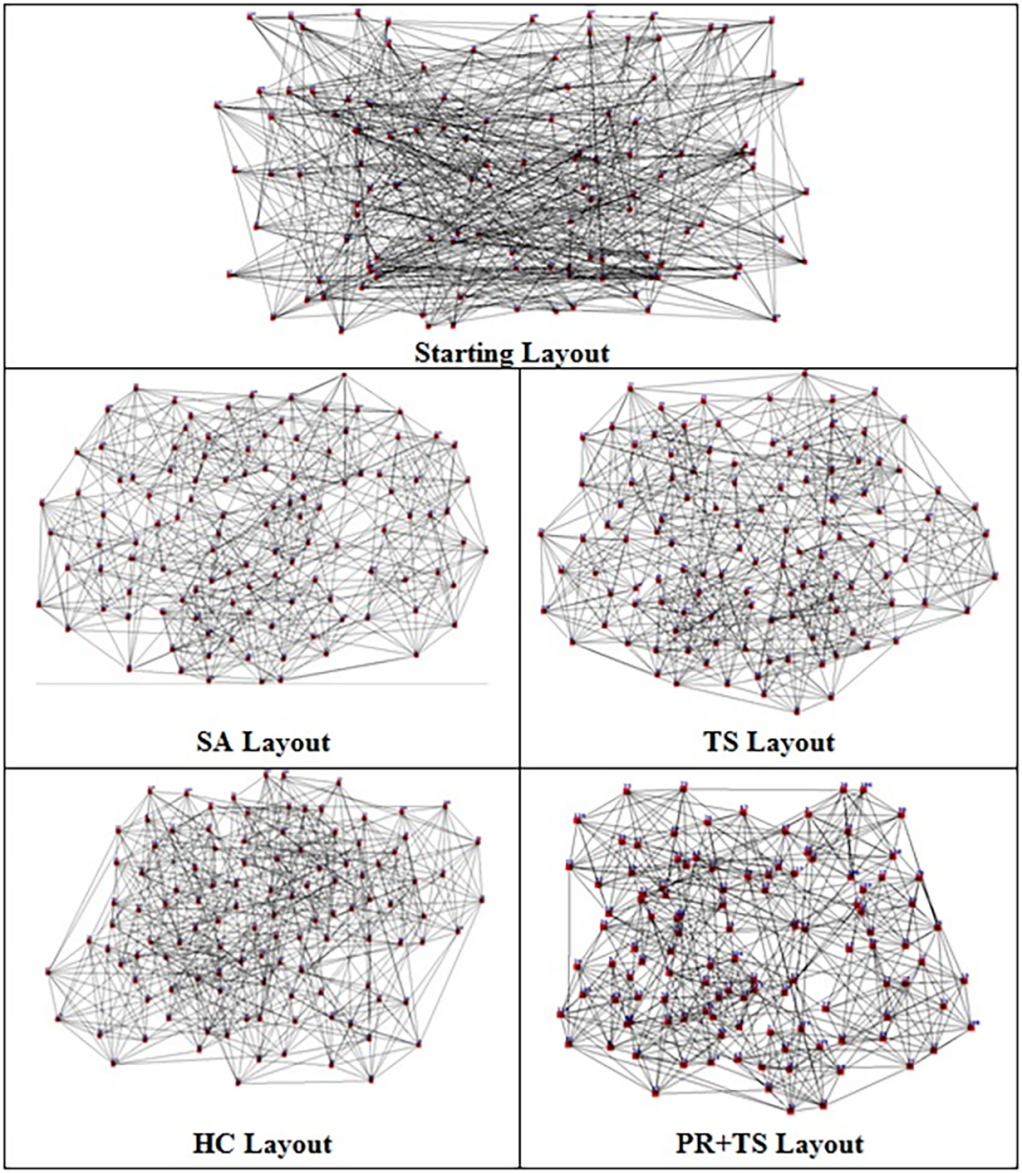
Layouts of graph dataset 5 (listed in Table 13) produced by all the methods.

Figs 31 and 32 are two examples of the layouts produced by all the methods when applied to graph 3 and graph 5 respectively in the list of real world datasets described in Table 13. We also report the normalized values of each aesthetic used in our objective function independently when the methods were applied on both graphs as shown in Table 14 and Table 15.

**Table 14:** Normalized values of each aesthetic when the methods were applied on graph dataset 3 (listed in Table 13).

	node-node occlusion	edge length	edge crossings	angular resolution
**HC**	0.029602	0.119400	0.075273	0.249458
**SA**	0.021657	0.084227	0.038497	0.230175
**TS**	0.026453	0.061855	0.038879	0.219136
**PR+TS**	0.000279	0.024855	0.024902	0.085991

**Table 15:** Normalized values of each aesthetic when the methods were applied on graph dataset 5 (listed in Table 13).

	node-node occlusion	edge length	edge crossings	angular resolution
**HC**	0.079880	0.164624	0.058610	0.395361
**SA**	0.035921	0.077498	0.033709	0.312177
**TS**	0.048987	0.096359	0.039349	0.346663
**PR+TS**	0.000156	0.031453	0.026176	0.195369

## 6. Analysis of results

Our graph drawing method of coupling tabu search with path relinking outperforms the other tested methods in terms of the quality of the produced graph layouts and number of evaluated solutions needed to reach a particular objective function’s value.

In experiment 1 we tested simulated annealing, hill climbing and tabu search. In experiment 2 we tested simulated annealing, tabu search and tabu search with path relinking. We covered three comparisons: phase I, diagram layouts with the best objective function’s value that can be achieved; phase II, number of evaluated solutions performed by each drawing algorithm to reach a particular level of layout quality; and phase III, quality of layout drawn by drawing algorithms after a fixed number of evaluated solutions.

When we were looking for a layout with the best objective function’s value that can be achieved, experiment 1 phase I, Fig 11 shows that simulated annealing produces graph layouts with the best objective function’s value compared to hill climbing and tabu search, with hill climbing the worst. On the other hand, simulated annealing evaluates a large number of solutions in order to get the layouts compared tabu search and Fig 12 shows that tabu search clearly outperforms the other two methods in terms of performance efficiency. In experiment 2 phase I, Fig 15 shows that tabu with path relinking outperforms the other methods in the quality of the layouts but with the highest number of evaluated solutions as shown in Fig 16.

In experiment 1 phase II, where we tested number of evaluated solutions performed by the drawing algorithms to reach a particular level of quality, see Fig 13 and Table 4, our tabu search method generates graph layouts of good quality with fewer number of evaluated solutions compared to hill climbing and simulated annealing. Calling the path relinking procedure within our tabu search procedure improves the performance of the drawing algorithm and reduces the number of evaluated solutions in experiment 2 phase II, see Fig 17 and Table 8. In experiment 2 phase II, see Table 10, it is clear that path relinking outperforms simulated annealing in drawing graph layouts with similar objective function’s values using a few number of evaluated solutions. It also outperforms the pure tabu search procedure on large graphs (as number of nodes increases) unlike smaller graphs where there is no significant difference.

Finally, for phase III, we ran the drawing algorithms so that they evaluate a specific number of solutions to test the quality of layouts that would be generated in a set time. For experiment 1 phase III we conclude from Fig 14 and Table 5 that our tabu search approach draws graph layouts with better quality (or similar quality in the worst case) compared to hill climbing and simulated annealing when they evaluate the same number of solutions. Adding path relinking to tabu search in experiment 2 phase III has improved the quality of the layouts compared to those layouts produced by pure tabu search procedure using the same number of evaluated solutions as shown in Fig 18 and Table 9. Table 11 showed that our tabu search/path relinking method draws graph layouts with better quality compared to simulated annealing. It also outperforms pure tabu search as the size of the graph increases, but there is no significant difference on smaller graphs.

The results of our experiments gives us strong evidence that our tabu search with path relinking outperforms hill climbing, simulated annealing, and pure tabu search procedures, and has a better scalability.

## 7. Conclusions

We have described a novel automated neighbourhood search method for drawing general graph layouts with undirected straight lines based on a weighted sum multi-criteria optimization. Our new method is based on tabu search with path relinking. The method searches for the best positions of the nodes, so minimizing the value of the objective function and drawing a nice graph layout. The integration of features of tabu search and path relinking in one implementation makes our method a more effective graph layout method than other well-known neighbourhood search methods such as hill climbing and simulated annealing. The key feature in tabu search is the combination of forbidding reverse moves using a memory-based tabu list and allowing escapes from local optima. Whereas building a reference set of elite solutions generated by tabu search and moving efficiently along the path between two solutions are the main aspects of our path relinking procedure. We also developed a systematic way for choosing the values of the parameters used by the method.

Our experimental results on random graphs and real world graphs show that our tabu search/path relinking approach draws graph layouts with good quality in a relatively low number of evaluated solutions. Coupling tabu search with path relinking outperforms simulated annealing and hill climbing in both terms of quality of layout and speed of layout.

In terms of future work, the performance of our method can be further improved by implementing a hybrid of path relinking and a Greedy Randomized Adaptive Search Procedure (GRASP). This combination has been previously applied efficiently in many applications with promising results [13]. In addition, more investigation can be performed on the effectiveness of our approach in comparison with force-directed approaches and other population based approaches that have been previously used in the field of graph drawing such as ant colony optimization [58]. Also, we can use a double-blind test on real human users to evaluate the layouts generated by different graph drawing algorithms as visualization is also concerned of how significant the differences are to human eye and the human sense of aesthetics. Finally, experiments can be conducted to study the efficiency of this method when applied to different types of graphs such as trees, hierarchical, and circular graphs. Our method can be easily adjusted to work with directed edges, but each type of these graphs has its own aesthetic measures such as: subtree separation, closest and farthest leaves for tree graphs; uniform edge direction and cycle removal for hierarchical graphs; partitioning the graph into clusters and placing the nodes of each cluster onto the perimeter of an embedding circle for circular graphs [59]. These aesthetics, in addition to the ones discussed in this paper which usually exist in any graph, must be formulated in a weighted sum multi-criteria objective function to be optimized by our proposed method.
